# Which are the most frequently involved peripheral joints in calcium pyrophosphate crystal deposition at imaging? A systematic literature review and meta-analysis by the OMERACT ultrasound – CPPD subgroup

**DOI:** 10.3389/fmed.2023.1131362

**Published:** 2023-03-09

**Authors:** Antonella Adinolfi, Silvia Sirotti, Garifallia Sakellariou, Edoardo Cipolletta, Emilio Filippucci, Francesco Porta, Anna Zanetti, Nicola Ughi, Piercarlo Sarzi-Puttini, Carlo Alberto Scirè, Helen Keen, Carlos Pineda, Lene Terslev, Maria Antonietta D’Agostino, Georgios Filippou

**Affiliations:** ^1^Rheumatology Division, Multispecialist Medical Department, ASST Grande Ospedale Metropolitano Niguarda, Milan, Italy; ^2^IRCCS Ospedale Galeazzi – Sant’Ambrogio, Rheumatology Department, Milan, Italy; ^3^Department of Internal Medicine and Therapeutics, Università di Pavia, Pavia, Italy; ^4^Istituti Clinici Scientifici Maugeri IRCCS, Pavia, Italy; ^5^Rheumatology Unit, Department of Clinical and Molecular Sciences, Polytechnic University of Marche, Ancona, Italy; ^6^Interdisciplinary Pain Medicine Unit, Rheumatology Section, Santa Maria Maddalena Hospital, Rovigo, Italy; ^7^SIR Epidemiology, Research Unit, Milan, Italy; ^8^Department of Biomedical and Clinical Sciences, Università degli studi di Milano, Milan, Italy; ^9^School of Medicine, University of Milano Bicocca, Milan, Italy; ^10^Medicine and Pharmacology Department, The University of Western Australia, Murdoch, Perth, WA, Australia; ^11^Rheumatology Department, Instituto Nacional de Rehabilitacion, Mexico, Mexico; ^12^Center for Rheumatology and Spine Diseases, Rigshospitalet, Copenhagen University, Copenhagen, Denmark; ^13^Rheumatology, Fondazione Policlinico Universitario “Gemelli”, IRCCS, Rome, Italy

**Keywords:** ultrasound, calcium pyrophosphate crystal deposition, conventional radiography, systematic review, CPPD, chondrocalcinosis

## Abstract

**Objectives:**

To identify the prevalence of calcium pyrophosphate crystal deposition (CPPD) using ultrasound and conventional radiology at peripheral joints in patients with suspected or definite CPPD.

**Methods:**

A systematic literature search was performed in PubMed and Embase using pre-defined search strategies from inception to April 2021 to identify studies that evaluated conventional radiology and ultrasound in detecting CPPD at peripheral joints, including definite or suspected CPPD [Research question 1 (RQ1) and Research Question 2 (RQ2), respectively]. For the meta-analysis, the first, second, and third sub-analysis included studies with the knee, and knee or wrist as the index joint for CPPD (without restrictions on the reference standard) and synovial fluid analysis or histology as a reference standard (without restrictions on the index joint), respectively.

**Results:**

One-thousand eight hundred and twenty-seven manuscripts were identified, of which 94 articles were finally included. Twenty-two and seventy-two papers were included in RQ1 and RQ2, respectively. The knee had the highest prevalence for RQ1 and RQ2 by both conventional radiology and ultrasound, followed by the wrist with the highest prevalence for RQ1. The hand had the lowest CPPD prevalence. The third sub-analysis showed a higher CPPD prevalence on ultrasound than conventional radiology at the knee (only data available).

**Conclusion:**

Among all peripheral joints, the knees and wrists could be regarded as the target joints for CPPD detection by imaging. Furthermore, ultrasound seems to detect a higher number of calcium pyrophosphate deposits than conventional radiology, even when using a more restrictive reference standard.

## Introduction

Calcium pyrophosphate deposition (CPPD) is a chronic arthropathy caused by the presence of calcium pyrophosphate (CPP) deposits in articular and periarticular tissues (1). Although the exact incidence and prevalence of CPPD are still unknown, it is considered one of the most common chronic arthropathies (2), characterized by a prevalence that increases with age (3) and can reach up to 13% in the elderly, depending on the assessed joints and the tool used (4). In fact, one of the main issues for epidemiological studies on CPPD is related to the challenges regarding diagnosis.

For a long time, CPPD diagnosis was based on McCarty Criteria, which required both the identification of CPP crystals in synovial fluid analysis (SFA) and the presence of typical calcifications in conventional radiography (CR) for a “definite” diagnosis, while a “probable” diagnosis was defined by SFA or CR positive findings (5). In 2011, a panel of experts from the European League against Rheumatism (EULAR) changed this status. Experts stated that the presence of CPP crystals in the SFA was sufficient for a definite diagnosis. Furthermore, ultrasonography (US) has been endorsed for the first time as a promising tool for CPPD diagnosis (6).

Since then, growing interest in the use of imaging in CPPD has led to an improved definition of the framework for CPPD diagnosis. In particular, US application in CPPD management has been highly improved since its development by the CPPD subgroup of the OMERACT US Working group of a new set of US definitions for CPPD identification (7, 8), which demonstrated the reliability and accuracy of CPPD diagnosis (8–10). A recent systematic literature review (SLR) evaluated the diagnostic performance of CR and US in CPPD diagnosis, showing that both obtained good results with better sensitivity for US and slightly greater specificity for CR (11). Furthermore, an international working group composed of rheumatologists and musculoskeletal radiologist experts in microcrystalline arthritis has recently developed definitions for CPPD identification by CR, which were also assessed for reliability and accuracy, confirming the high specificity of CR for CPPD identification (12, 13).

Accounting for all, the use of imaging is gaining a leading role in CPPD diagnosis and potentially for follow-up in daily practice. However, given the wide range of CPPD joint involvement, it is particularly important to adopt a time-saving approach for US examination by assessing only the most frequently affected peripheral joints, thus increasing the effectiveness and feasibility. The identification of a minimum set of joints could also promote the application of a scoring system, which could be very useful in monitoring the evolution of CPPD.

Thus, the objective of this study was to perform a SLR to estimate the prevalence of CPPD, identified using CR, US or both at the peripheral joints of patients with a suspected or definite CPPD diagnosis, and to establish the most relevant joints for CPPD diagnosis and monitoring.

## Methods

The Preferred Reporting Items for Systematic Reviews and Meta-analyzes (PRISMA 2020) guidelines for reporting systematic reviews and meta-analyzes were followed for this review (14).

A protocol defining all phases of this SLR (research questions, search strategy, and inclusion/exclusion criteria for the articles and methods for the analysis) was developed before the beginning of the study and was registered on the PROSPERO platform (Registration Number: CRD42020218155).

### Structured search strategy

Two research questions were developed; the first aimed to assess the CPPD prevalence in peripheral joints based on imaging of patients with a definite, crystal proven, CPPD diagnosis [Research Question 1 (RQ1)], and the second one aimed to assess the prevalence of CPP deposits, based on imaging, in patients with suspicion of CPPD diagnosis according to clinical picture [Research Question 2 (RQ2)].

After defining the research questions, the patient, intervention, comparator, outcome (PICO) framework was used to develop the search strategy (15).

PubMed and Embase databases were searched from inception until April 2021. An additional hand search of articles’ references was performed to include as many eligible articles as possible. The search strategy was based on both MeSH terms and free text and is illustrated in the Supplementary material S1 (SP1).

### Study selection and data extraction

The search included all the studies that evaluated the use of CR and/or US for detecting calcifications at the level of at least one peripheral joint [hand, wrist, elbow, shoulder, acromioclavicular (AC), hip, knee, ankle, foot] in adult patients with suspected or definite CPPD, without any restrictions on the reference test used for diagnosis.

The following study types were eligible for inclusion: cross-sectional case–control, cross-sectional cohort, longitudinal case–control, longitudinal cohort, retrospective cohort, and retrospective case–control. Case reports, case series, congress abstracts, and studies written in languages other than English were excluded.

The titles and abstracts of the retrieved references were screened by six reviewers (AA, EC, EF, GF, FP, and SS) according to pre-defined inclusion and exclusion criteria based on the PICOs. The reviewers worked in pairs to assess the abstracts, and discordant assessments were resolved by consensus.

Relevant full-text articles were evaluated by the same reviewers, and data were extracted using a standardized extraction form. Discordant assessments between the authors were resolved by consensus. Data were extracted using a standardized form, including author, publication year, study type, index test, reference test, inclusion criteria, and number of patients (cases and controls). The data on the frequency of calcifications are summarized in *ad hoc* tables.

For each article, data on the prevalence of calcifications were collected separately for every peripheral joint, according to the imaging technique applied. If available, data on the involvement of single joint structures (fibrocartilage, hyaline cartilage, tendons) were also retrieved. The frequency of involvement was assessed separately for every joint and according to the imaging techniques used. For each joint and structure, data of monolateral or bilateral involvement of calcifications were collected, divided according to the imaging tool. In case of missing laterality data, they were categorized as unknown.

### Assessment of the risk of bias

The risk of bias of the selected studies was assessed using *ad hoc* instruments applied according to the type of article evaluated. For the diagnostic study, we used the modified version of the Quality Assessment of Diagnostic Accuracy Studies (QUADAS-2) tool (16), while and the Newcastle Ottawa Scale (NOS) was used for the assessment of case–control and cohort studies (17). Data extraction and quality assessment were performed by a single reviewer (EC) and checked by a second reviewer (SS). Any disagreements were resolved by consensus.

### Data analysis

Descriptive analyzes and meta-analyzes were performed on the included studies. The descriptive analysis aimed to capture the global prevalence of CPPD in different joints. Except for a relevant degree of variability in the included studies regarding the index joints, reference standards, and imaging techniques, specific meta-analyzes were scheduled to collect as much data as possible from homogenous studies. Thus, the following analyzes were performed:

- Descriptive analysis including all studies: Evaluation of CPP deposit prevalence for each joint assessed. All analyzes were divided according to the research question and the imaging method used. If available in the text, the frequency of bilateral involvement at each anatomical site was also provided.
- Descriptive analysis including all studies: evaluation of CPP deposit prevalence at the level of the anatomical structures of a single joint. All analyzes were divided according to the research question and the imaging method used. If available, the frequency of bilateral involvement was also provided.
- Sub-analysis 1 (SB_1) included only studies that used the knee as the index joint for CPPD diagnosis, independent of the reference standard used. All analyzes were divided according to the research question and the imaging method used.
- Sub-analysis 2 (SB_2) included studies that used the knee or wrist as the index joint for CPPD diagnosis, independent of the reference standard used. All analyzes were divided according to the research question and the imaging method used.
- Sub-analysis 3 (SB_3) included only studies that used SFA alone (not the McCarty criteria) or histology as a reference standard for the diagnosis of CPPD independently from the index joint and imaging method used. All analyzes were divided according to the research question and the imaging method used.

SB_1 and SB_2 aimed to assess the impact of the index joint, and SB_3 aimed to assess the impact of the reference test on CPP deposit prevalence.

### Statistical analysis

The descriptive analyzes were provided as the ratio between the imaging-positive cases and all the cases evaluated (either for the joint or the single articular structure). The results were also presented as percentages. About the bilateral assessment, the results were obtained evaluating all the imaging cases bilaterally positive and the cases evaluated bilaterally (shown in the text as ratios and percentages).

For the sub-analysis, 162 meta-analyzes were performed, one referring to each research question, sub-analysis, and joint analysis. Only meta-analyzes that included at least three studies were considered and presented in this paper. Information on the proportion of participants with CPP deposits in different joints was extrapolated from each study. Pooled estimates [with related 95% confidence intervals (CI)] were calculated using both fixed- and random-effects models. Heterogeneity was calculated using the I^2^ index and was high in all analyzes. For this reason, only pooled estimates from random-effects models were reported in the results section. The results were graphically presented using forest plots. All analyzes were performed using the R statistical software (Foundation for Statistical Computing, Vienna, Austria).

## Results

### Description of the studies

The search strategy identified 1827 records, 1822 from the databases, and five manually searched records (329 duplicates). Of the remaining 1,498 records, 954 were excluded based on their titles and abstracts, and 544 articles entered the full-text evaluation. Considering that 49 full texts were not retrievable (all articles were published before the 1970s), the detailed review included 494 articles. A total of 400 studies were excluded after reviewing the full text, most of which were rejected due to the study type, mainly case reports and case series, followed by outcome. Finally, 94 studies were included in the analysis.

**Figure 1:**
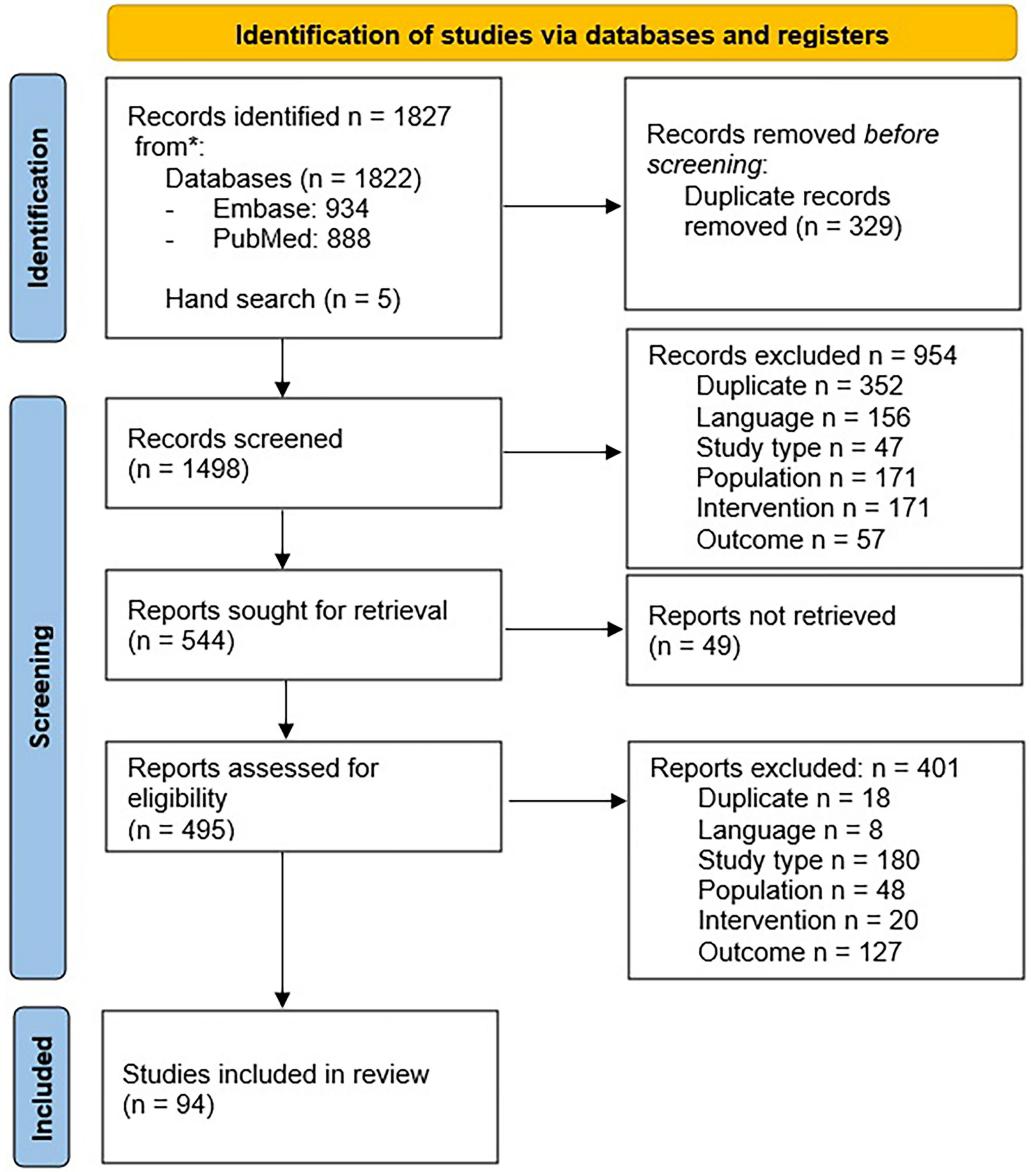
PRISMA 2020 flow diagram for systematic reviews.

All phases of the selection process are summarized in the Prisma Flow Chart (Figure 1).

For RQ1, 22 papers (18–39) were included, with a total population of 1,425 patients, 876 cases, and 549 controls. One study satisfied both research questions (34), including 16 patients with a definite CPPD diagnosis and 27 with a suspected diagnosis, using CR as either an index or a reference test.

The imaging technique used to detect the calcific deposits was CR alone in 10 articles (19, 29, 31–38) and US in four articles (23, 25–27), while eight papers evaluated both CR and US (18, 20–22, 24, 28, 30, 39). The reference standards were the McCarty Criteria in 12 articles (19–26, 29, 35, 36, 39), and SFA and CR alone in two (30, 38) and six (27, 31–34, 37)papers, respectively.

In RQ2, 72 articles were included (34, 40–110) with a total population of 30,480 patients, 3,027 cases, and 27,453 controls.

The imaging technique applied for the calcifications was CR in 61 papers (2, 38–53, 55–57, 59–63, 68–78, 80–88, 91–96, 98–107) and US alone in six studies (56, 60, 66, 68, 92, 110), while in four articles both imaging techniques (67, 69, 81, 99) were used. The reference standard applied for CPPD diagnosis was CR in 53 articles (34, 40, 41, 43, 45, 47–51, 53–55, 57–59, 61–65, 70–78, 80, 82–85, 87–90, 93, 94, 96–98, 100–105, 107–109), the McCarty criteria were used in five papers (42, 44, 46, 60, 95), US in four (56, 66, 69, 92) and SFA in eight studies (52, 68, 79, 81, 91, 99, 106, 110). Finally, only two articles used histology as reference standard (67, 86).

Considering both RQ1 and RQ2, the most assessed joint was the knee included in 74 papers (18, 19, 25–27, 29, 30, 33–35, 37–40, 42–51, 54–56, 58–76, 78, 80–85, 87–91, 93–102, 105–110), mainly as a single joint examined (39/72 papers), followed by the wrist included in 43 papers (19–21, 25, 32–37, 40, 42, 44–47, 51, 53, 56, 57, 59, 61, 69–73, 82, 83, 85, 87, 93, 95–97, 100, 102–105, 107, 108, 110) and the hip in 18 studies (22, 35, 37, 40–42, 45, 46, 59, 73, 77, 78, 85–87, 97, 102, 107), while the less included was the AC, evaluated only in four articles (31, 35, 92, 94). The characteristics of all included studies are summarized in Table 1, and all the data of each article assessed are summarized in SP2.

**Table 1:** Characteristics of the articles entered in the SLR.

Article	Study type	Population	No of patients	Imaging technique	Reference standard	Joint assessed
			(Cases/controls)			
Definite CPPD diagnosis (RQ1)						
Barskova et al., 2013 (18)	Cross-sectional cohort	CPPD	25	US, CR, CT	SFA	Knee
Canhao et al., 2001 (19)	Cross-sectional cohort	CPPD	50	CR	McCarty criteria	Knee, Wrist
Cipolletta et al., 2020 (20)	Cross-sectional case–control	Cases: CPPD, controls: other rheumatic diseases (RA, PsA, OA, SS, PMR, septic arthritis)	100 (61/39)	US, CR, CT	McCarty criteria	Wrist
Di Matteo et al., 2017 (21)	Cross-sectional case–Control	Cases: CPPD, controls: other rheumatic diseases (RA, PsA, OA, SA, SLE, gout, reactive arthritis)	84 (36/48)	US, CR	McCarty criteria	Wrist
Di Matteo et al., 2019 (22)	Cross-sectional case–control	Cases: CPPD, controls: other rheumatic diseases (OA, PsA, RA, SA, PMR, gout, SLE)	90 (50/40)	US, CR	McCarty criteria	Hip
Ellaban et al., 2012 (23)	Cross-sectional case–control	Patients with knee effusion available for aspiration	60 (38/22)	US	McCarty criteria	Ankle
Falsetti et al., 2004 (24)	Cross-sectional case–control	Cases: CC, controls: OA, HS	157 (57/100)	US, CR	McCarty criteria	Ankle
Filippou et al., 2013 (25)	Cross-sectional cohort	CPPD	42	US	McCarty criteria	Knee, wrist, hand, ankle
Filippucci et al., 2009 (26)	Cross-sectional case–control	Cases: CPPD, controls: gout, RA, PsA, OA	132 (48/84)	US	McCarty criteria	Knee
Foldes K, 2002 (27)	Cross-sectional case–control	Cases: CC, controls: OA	40 (21/19)	US	CR	Knee
Forien M et al., 2017 (28)	Cross-sectional case–control	Cases: CPPD, controls: patients without CPP crystals in SF	58 (32/26)	US, CR	SFA	Wrist
Gerster JC et al., 1977 (29)	Cross-sectional case–control	Cases: CC, controls: OA	104 (52/52)	CR	McCarty criteria	Knee, Ankle
Gutierrez M et al., 2014 (30)	Cross-sectional case–control	Cases: CPPD, controls: other rheumatic diseases (OA, SpA, RA, gout)	157 (74/83)	US, XR	SFA	Knee
Huang HS et al., 1993 (31)	Retrospective cohort	CPPD	53	CR	CR	Shoulder, AC
Linden et al., 1977 (32)	Cross-sectional cohort	CC	22	CR	CR	Wrist, hand
Moskowitz RW et al., 1967 (33)	Cross-sectional cohort	CC	24	CR	CR	Knee. Wrist, hand, elbow, shoulder, hip, ankle
Peter et al. 2001# (34)	Cross-sectional cohort	CC, OA	16	CR	CR	Wrist
Resnick et al., 1977 (35)	Retrospective cohort	CPPD	85	CR	McCarty criteria	Knee, wrist, hand, elbow, shoulder, hip, ankle, foot, AC
Resnick et al., 1974 (36)	Retrospective cohort	CPPD	18	CR	McCarty criteria	wrist
Richardson et al., 1983 (37)	Cross-sectional cohort	Familiar CPPD	11	CR	CR	Knee, wrist, hand, shoulder, hip, ankle, foot
Schlesinger et al., 2009 (38)	Retrospective cohort	CPPD	67	CR	SFA	Knee
Vele et al., 2018 (39)	Cross-sectional cohort	CPPD	30	CR, US	McCarty criteria	Knee, wrist, shoulder, AC, hip, ankle
Suspected CPPD diagnosis (RQ2)						
Abhishek et al., 2012 (40)	Cross-sectional cohort	OA	3,118 (428/2690)	CR	CR	Knee, wrist, hand, hip
Axford et al., 1991 (41)	Cross-Sectional cohort	HHC	112 (17/95)	CR	CR	Hip
Balsa et al., 1990 (42)	Cross-sectional cohort	Familiar CPPD	175 (46/129)	CR	McCarty criteria	Knee, wrist, shoulder, hip
Béija et al., 2004 (43)	Cross-sectional cohort	Familiar CPPD	103 (15/88)	CR	CR	Knee
Bergstrom et al., 1986 (44)	Longitudinal cohort	Pt > 70	352 (37/315)	CR	CR	Knee, wrist, hand
Bjelle et al., 1982 (45)	Cross-sectional cohort	Familiar CPPD	30 (21/9)	CR	CR	Knee, wrist, elbow, shoulder, hip, ankle,
Bjelle et al., 1974 (46)	Cross-sectional cohort	Pts with knee pain	300 (50/250)	CR	McCarty criteria	Knee, wrist, hip
Brasseur et al., 1987 (47)	Cross-sectional cohort	RA	100 (3/97)	CR	CR	Knee, wrist
Chaisson et al., 1996 (48)	Cross-sectional cohort	OA	1,416 (114/1302)	CR	CR	Knee
Chiba et al., 2018 (49)	Longitudinal cohort	HS	1,278 (28/1250)	CR	CR	Knee
Cho et al., 2018 (50)	Longitudinal cohort	HS	4,543 (121/4422)	CR	CR	Knee
De la Garza et al., 2019 (51)	Retrospective cohort	Pt > 50 years old	1,602 (47/1555)	CR	CR	Knee, wrist
Derfus et al., 2002 (52)	Cross-sectional cohort	Pt undergoing TKR	53 (16/37)	CR	SFA	Knee
Devauchelle-Pensec et al., 2006 (53)	Longitudinal case–control	Pt affected by arthritis ≤1 year last	258 (5/253)	CR	CR	Wrist
Doherty et al., 1996 (54)	Longitudinal cohort	OA	135 (43/92)	CR	CR	Knee
Doherty et al., 1982 (55)	Cross-sectional case-contro	Pt submitted to unilateral meniscectomy	200 (23/177)	CR	CR	Knee
Ellabban et al., 2012 (56)	Cross-sectional case–control	Pt with knee effusion	60 (32/28)	US	US	Knee, wrist
Ellman et al., 1979 (57)	Retrospective case–control	Pt undergoing long-term hemodialysis	82 (3/79)	CR	CR	Wrist
Ellman et al., 1981 (58)	Retrospective cohort	Pt older than 50 with knee CR	574 (55/519)	CR	CR	Knee
Ellman et al., 1975 (59)	Cross-sectional cohort	Volunteers among ambulatory residents	58 (16/42)	CR	CR	Knee, wrist
Falsetti et al., 2011 (60)	Longitudinal case–control	PMR	61 (9/52)	US	McCarty criteria	Knee, Ankle
Faraawi et al., 1993 (61)	Cross-sectional cohort	HHC	25 (9/16)	CR	CR	Knee, wrist
Feller et al., 1972 (62)	Cross-sectional case–control	Wilson’s disease	17 (2/15)	CR	CR	Knee
Felson et al., 1989 (63)	Longitudinal cohort	OA	1,402 (114/1288)	CR	CR	Knee
Felson et al., 1997 (64)	Longitudinal case–control	OA	979 (84/895)	CR	CR	Knee
Fernandez Dapica et al., 1986 (65)	Cross-sectional Cohort	Family members older than 13 of pt. affected by primary CPPD	149 (19/130)	CR	CR	Knee
Filippou et al., 2007 (66)	Cross-sectional case–control	Cases: CPPD, controls: joint effusion without CPPD	43 (14/29)	US	US	Knee
Filippou et al., 2016 (67)	Cross-sectional cohort	OA (waiting for TKR)	42 (26/16)	US, CR	Hystology	Knee
Filippou et al., 2020 (68)	Cross-sectional cohort	Pt > 55 with knee pain and swelling	67 (42/25)	US	SFA	Knee
Frediani et al., 2005 (69)	Cross-sectional case–control	Suspected CPPD	24 (11/13)	US, CR	US, SFA	Knee, wrist
Good et al., 1967 (70)	Retrospective case–control	Gout, RA	81 (8/73)	CR	CR	Knee, wrist, elbow, shoulder
Gordon et al., 1984 (71)	Cross-sectional Cohort	Pt older than 50	127 (20/107)	CR	CR	Knee, wrist
Hamilton EBD et al., 1981 (72)	Longitudinal cohort	HHC	18 (13/5)	CR	CR	Knee, wrist, hip
Hamza et al., 1992 (73)	Longitudinal cohort	CC	77 (7/70)	CR	CR	Knee, wrist, hand, elbow, shoulder, hip, ankle, foot
Hernborg et al., 1977 (74)	Longitudinal cohort	OA	84 (22/62)	CR	CR	Knee
Komatireddy et al., 1989 (75)	Cross-sectional case–control	Cases: HT, controls: HS	80 (3/77)	CR	CR	Knee
Latourte et al., 2020 (76)	Retrospective cohort	OA	656 (93/563)	CR	CR	Knee
Ledingham et al., 1992 (77)	Cross-sectional cohort	OA (hip)	211 (23/188)	CR	CR	Hip
Ledingham et al., 1993 (78)	Longitudinal cohort	OA (hip)	136 (13/123)	CR	CR	Hip
Ledingham et al., 1993 (79)	Cross-sectional cohort	OA (knee)	252 (132/120)	CR	SFA	Knee
Ledingham et al., 1995 (80)	Cross-sectional cohort	OA (knee)	188 (62/126)	CR	CR	Knee
Lee et al., 2019 (81)	Cross-sectional cohort	Knee effusion	174 (43/131)	CR, US	SFA	Knee
Massardo et al., 1989 (82)	Longitudinal cohort	OA	31 (9/22)	CR	CR	Knee, wrist
Mathews et al., 1987 (83)	Retrospective cohort	HHC	45 (3/42)	CR	CR	Knee, wrist
McAlindon et al., 1996 (84)	Cross-sectional cohort	OA	600 (94/506)	CR	Cr	Knee
Menerey et al., 1988 (85)	Cross-sectional cohort	Wilson’s Disease	22 (3/19)	CR	CR	Knee, wrist, shoulder, hip
Montgomery et al., 1998 (86)	Longitudinal cohort	HHC	15 (4/11)	CR	Hystology	Hip
Musacchio et al., 2011 (87)	Cross-sectional cohort	Pts older than 65	1,629 (169/1460)	CR	CR	Knee, Hip
Neame et al., 2003 (88)	Cross-sectional Cohort	Pts f a community-based study	1727 (119/1608)	CR	CR	Knee
Neogi et al. (BOKS) 2006* (89)	Longitudinal cohort	OA	265 (23/242)	CR	CR	Knee
Neogi et al. (HEALTH ABC), 2006* (89)	Longitudinal cohort	African American and white adults, ages 70–79 years	230 (69/161)	CR	CR	Knee
Nguyen et al., 2013 (90)	Cross-sectional cohort	Pts undergoing TKR	20 (4/16)	CR	CR	Knee
Ottaviani et al., 2015 (91)	Cross-sectional cohort	Knee effusion	51 (25/26)	CR, US	SFA	Knee
Ottaviani et al., 2020 (92)	Cross-sectional case–control	PMR	75 (29/46)	US	US	AC
Paalanen et al., 2020 (93)	Longitudinal cohort	RA	435 (17/418)	CR	CR	Knee, wrist, shoulder, foot
Parperis et al., 2013 (94)	Retrospective cohort	Pts older than 50	1920 (78/1842)	CR	CR	Knee, AC
Pego-Reigosa et al., 2005 (95)	Longitudinal case–control	PMR, CPPD	118 (36/82)	CR	McCarty criteria	Knee, wrist
Peter et al., 2001# (34)	Longitudinal cohort	1^st^ CMC OA	27 (2/25)	CR	CR	Wrist, Knee
Pritchard et al., 1977 (96)	Cross-sectional cohort	Pts submitted to postparathyroidectomy or admitted to the acute geriatric unit	141 (24/117)	CR	CR	Knee, wrist
Reginato et al., 1976 (97)	Cross-sectional cohort	Pts with rheumatic symptoms	208 (36/172)	CR	CR	Knee, wrist, hand, elbow, shoulder, hip, ankle, foot
Richette et al., 2007 (98)	Cross-sectional case–control	Cases: pts. receiving HPN	144 (14/130)	CR	CR	Knee
		Controls: age- and sex-matched subjects				
Ruta et al., 2016 (99)	Cross-sectional cohort	Patients ≥50 years old with knee effusion	75 (15/60)	CR, US	SFA	Knee
Sanmarti et al., 1993 (100)	Retrospective cohort	Pts older than 60	261 (27/234)	CR	CR	Knee, wrist, hand
Schouten et al., 1992 (101)	Longitudinal cohort	Pts born after 1909 with OA	142 (13/129)	CR	CR	Knee
Stockman et al., 1980 (102)	Cross-sectional case–Control	Cases: gout, controls: volunteers without gout	280 (8/272)	CR	CR	Knee, wrist, hip
Trentham et al., 1975 (103)	Cross-sectional cohort	HS	100 (2/98)	CR	CR	Wrist
Utsinger et al, 1975 (104)	Cross-sectional cohort	Wrist arthropathy	18 (12/6)	CR	CR	Wrist
van der Korst et al., 1974 (105)	Cross-sectional cohort	Relatives of pts. affected by CPPD (familiar form)	108 (22/86)	CR	*CR*	Knee, wrist, hand, shoulder
Viriyavejkul et al., 2007 (106)	Cross-sectional cohort	OA	102 (53/49)	CR	SFA	Knee
Wilkins et al., 1983 (107)	Cross-sectional cohort	Consecutive OA patients	100 (34/66)	CR	CR	Knee, wrist, hand, hip
Yashiro et al., 1991 (108)	Cross-sectional Cohort	PHPT	132 (8/124)	CR	CR	Knee, wrist
Zhang et al., 2004 (109)	Cross-sectional case-contro	Siblings of pts. with CPP arthropathy	1843 (134/1709)	CR	CR	Knee
Zufferey et al., 2015 (110)	Cross-sectional cohort	Consecutive patients who presented with acute arthritis	109 (37/72)	US	SFA	Knee, wrist, hand, ankle, foot

Legend: Pts-patients, CPPD-calcium pyrophosphate deposition disease, CPP-calcium pyrophosphate, CC-chondrocalcinosis, US-ultrasound, CR-conventional radiography, CT-computed tomography, MRI-Magnetic Resonance Imaging, DECT-Dual energy computed tomography, AC: acromion-clavicular, SF-synovial fluid, SFA-synovial fluid analysis, RA-Rheumatoid Arthritis, PsA-psoriatic arthritis, SpA-spondyloarthritis, PMR-polymyalgia rheumatica, OA-osteoarthritis, SLE-systemic lupus erythematosus, SS-systemic sclerosis, HS-Healthy subjects, PHPT-hyperparathyroidism, HHC-hereditary hemochromatosis, TKR total knee replacement, JIA-Juvenile Idiopathic Arthritis, EHOA-erosive hand osteoarthritis, HT: hypothyroidism, PBC-primary biliary cirrhosis, HPP-Hypophosphatasia, CMC-carpo-metacarpal joint, HPN-home parenteral nutrition-PIN-posterior interosseous nerve. *This article includes the results of two different studies (the BOKS, a prospective natural history study of symptomatic knee OA, and the Health, Aging, and Body Composition-HEALTH ABC, a prospective cohort study). #This article was included in both research questions.

### Frequency of involvement of peripheral joints

#### Calcifications at imaging-descriptive results

Regarding RQ1, the wrist showed the highest CPP deposit prevalence, i.e., 92% (158/171) at US and 70% (240/343) at CR. A slightly lower prevalence was reported for the knee, i.e., 88% (146/166) at US and 62% (211/388) at CR. Regarding the other joints, the hip had the 65% (84/130) of deposit prevalence at CR and 90% (45/50) at US, while the prevalence at CR was 44% (34/77) and 31% (34/111) for elbow and shoulder, respectively. The lowest values were reported for the hand, with 19% (23/123) at CR and 9% (4/42) at US. Data on the laterality of joint involvement are not available for all the joints. The highest bilateral involvement was observed in the knee and wrist, with values of 86% (83/96) and 73% (49/67), respectively.

For RQ2, the knee was the joint with the highest CPPD prevalence, i.e., 85% (2,342/2770) and 93% (235/254) on CR and US, respectively, followed by the wrist with values that vary from 51% (492/955) at CR to 38% (27/80) at US. Considering only the sites assessed on a larger number of patients (>100), the hip and shoulder had a prevalence of CPP deposits equalling 36% (312/873) and 47% (68/143) on CR, respectively, while the hand showed the lowest prevalence, i.e., 14% (90/591) at CR. Bilateral involvement, mainly evaluable on CR, was higher in the wrist, knee, and hip, with values of 66% (203/309), 65% (641/985), and 50% (97/194), respectively.

All the descriptive results regarding joint involvement are summarized in Table 2.

**Table 2:** Calcifications prevalence at level of each joint assessed: overall results.

Definite CPPD Diagnosis (RQ1)										
		KNEE	WRIST	HAND	ELBOW	SHOULDER	AC	HIP	ANKLE	FOOT
CR	Imaging positive cases/all cases	211/338	240/343	23/123	34/77	34/111	33/79	84/130	22/132	15/59
		62%	70%	19%	44%	31%	42%	65%	17%	25%
	Cases positive bilaterally	41/70	36/64	NA	1/21	NA	9/17	29/43	2/52	NA
		58%	56%		5%		53%*	67%*	4%	
US	Imaging positive cases/all cases	146/166	158/171	4/42 (2)	NA	NA	NA	45/50	78/137	NA
		88%	92%	9%				90%*	57%	
	Cases positive bilaterally	83/96	49/67	1/4	NA	NA	NA	NA	38/56	NA
		86%	73%	25%					68%	
Suspected CPPD diagnosis (RQ2)										
		KNEE	WRIST	HAND	ELBOW	SHOULDER	AC	HIP	ANKLE	FOOT
CR	Imaging positive cases/all cases	2342/2770	492/955	90/591	29/61	68/143	21/78	312/873	22/52	22/45
		85%	51%	14%	47%	47%	27%	36%	42%	49%
	Cases positive bilaterally	641/985	203/309	60/84	26/26	43/49	NA	97/194	20/20	17/18
		65%	66%	71%	100%*	88%		50%	100%	94%
US	Imaging positive cases/all cases	235/254	27/80	1/37	NA	3/11	29/29	NA	2/37	8/37
		93%	38%	3%		27%	100%		5%*	22%*
	Cases positive bilaterally	NA	NA	NA	NA	NA	NA	NA	NA	NA

NA: Not Applicable (data not available). *value obtained by a single study.

### Frequency of involvement of the joint structures

Among the studies of RQ1, the joint structures characterized by the highest prevalence of calcific deposits were the menisci, i.e., 90% (67/74) on US, and 59% (172/292) on CR, followed by the triangular fibrocartilage of the wrist (TFC), 56% (70/126) and 47% (139/293) at US and CR, respectively; the knee hyaline cartilage, 66% (94/143) and 33% (80/242)at US and CR, respectively; and the hip fibrocartilage, 50% (45/90) on US 38% (32/85) on CR. Regarding laterality, some results were available for TFC, characterized by bilateral involvement of up to 87% (27/31) on CR and 67% (47/70) on US.

For RQ2, the values were higher for the TFC, followed by the menisci and hyaline cartilage, but only on US (30, 24, and 15%, respectively), while the results were lower at CR (5, 9, and 6%, respectively). Regarding laterality, higher bilateral involvement was recorded for the menisci at CR (28%).

All results regarding CPPD prevalence of joint structures and laterality are shown in SP3.

### Meta-analysis

In the SB_1, 73 articles (18, 19, 25–27, 29, 30, 33, 35, 37–40, 42–51, 54–56, 58–76, 78, 80–85, 87–91, 93–102, 105–110) (12 RQ1/61 RQ2) were included; 83 (18–21, 25–27, 29, 30, 32–40, 42–51, 54–56, 58–76, 78, 80–85, 87–91, 93–110) in the SB_2 (18 RQ1/65 RQ2); 14 papers (18, 28, 30, 38, 52, 67, 68, 79, 81, 86, 91, 99, 106, 110) (4 RQ1/10 RQ2) in the SB_3.

For RQ1, for both SB_1 and SB_2, the knee had an overall prevalence of 0.85 [0.62–0.95] with a higher value for US with respect to CR [0.93 (0.68–0.99) and 0.79 (0.44–0.95), respectively]. Regarding the wrist, the prevalence changed between SB_1 and SB_2. In fact, in SB_1, the overall CPP prevalence was 0.72 [0.47–0.88] with a lower value at CR 0.64 [0.39–0.83] and no data available in the US, while in SB_2 the overall prevalence was 0.87 [0.74–0.94], but the prevalence was higher for the US with respect to CR [0.92 (0.87–0.96) and 0.83 (0.61–0.94), respectively]. Decreasing overall prevalence values have been reported for the shoulders, ankles, and hands. The last was the joint characterized by the lowest result. Insufficient data were available for the other sites and SB_3.

With respect to RQ2, the knee had the highest prevalence without any differences between SB_1 and SB_2 and the imaging technique applied [overall value 0.98 (0.96–0.99), US 0.98 (0.88–1.00), CR 0.98 (0.96–0.99)]. Considering the wrist, the overall prevalence in SB_2 was 0.56 [0.45–0.66], with a greater prevalence on CR than on US [0.58 (048–0.68) and 0.33 (0.13–0.63) respectively]. For the other joints, the overall prevalence varied from 0.41 (0.10–0.81) for the elbow to 0.18 (0.11–0.29) for the hand. Considering SB_3, the data were available only for the knee, showing a higher prevalence when US was applied [overall value 0.87 (0.62–0.97), US 0.98 (0.75–1.00), CR 0.63 (0.35–0.84)].

**Figure 2:**
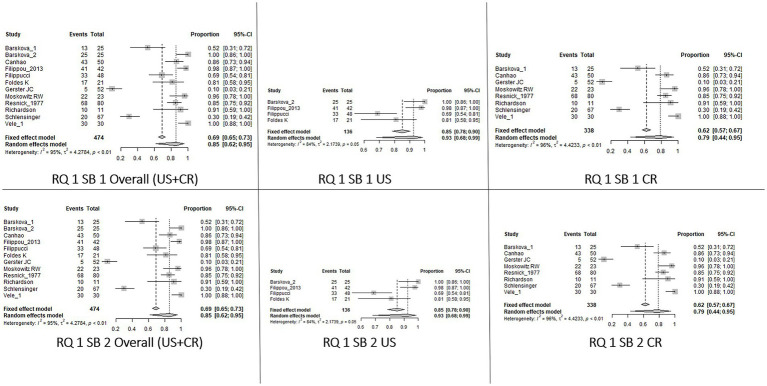
Forest Plot Knee, Research question 1 (RQ1), Sub_analysis 1 and 2: patients with definite diagnosis of CPPD and knee as index joint (SB_1) or knee and/or wrist (SB2) analyzed by imaging.

**Figure 3:**
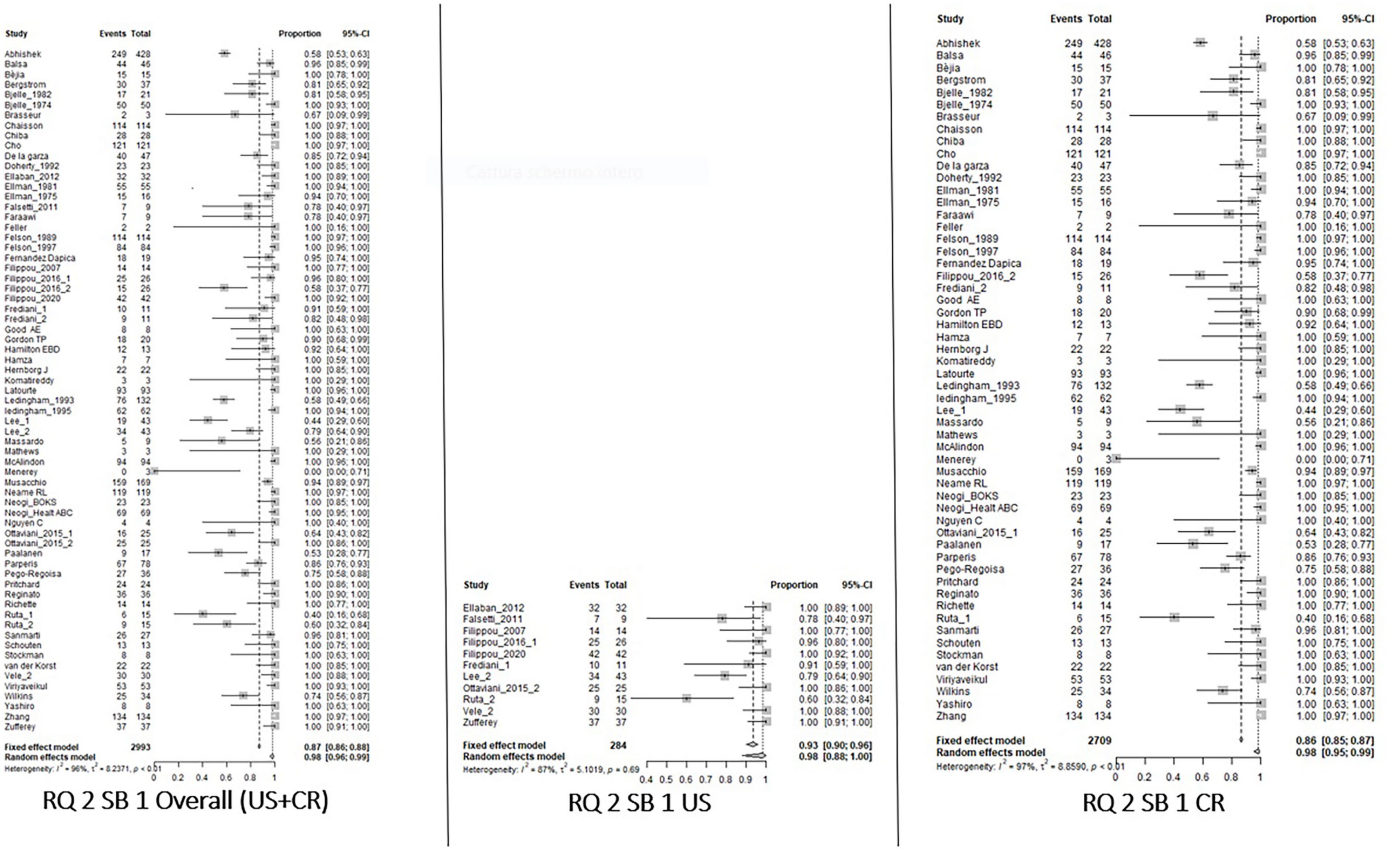
Forest Plot Knee, Research Question 2 (RQ 2), Sub_analysis 1: patients with suspected CPPD and the knee joint used as index joint, independently of the reference standard used for diagnosis, analyzed by imaging.

**Figure 4:**
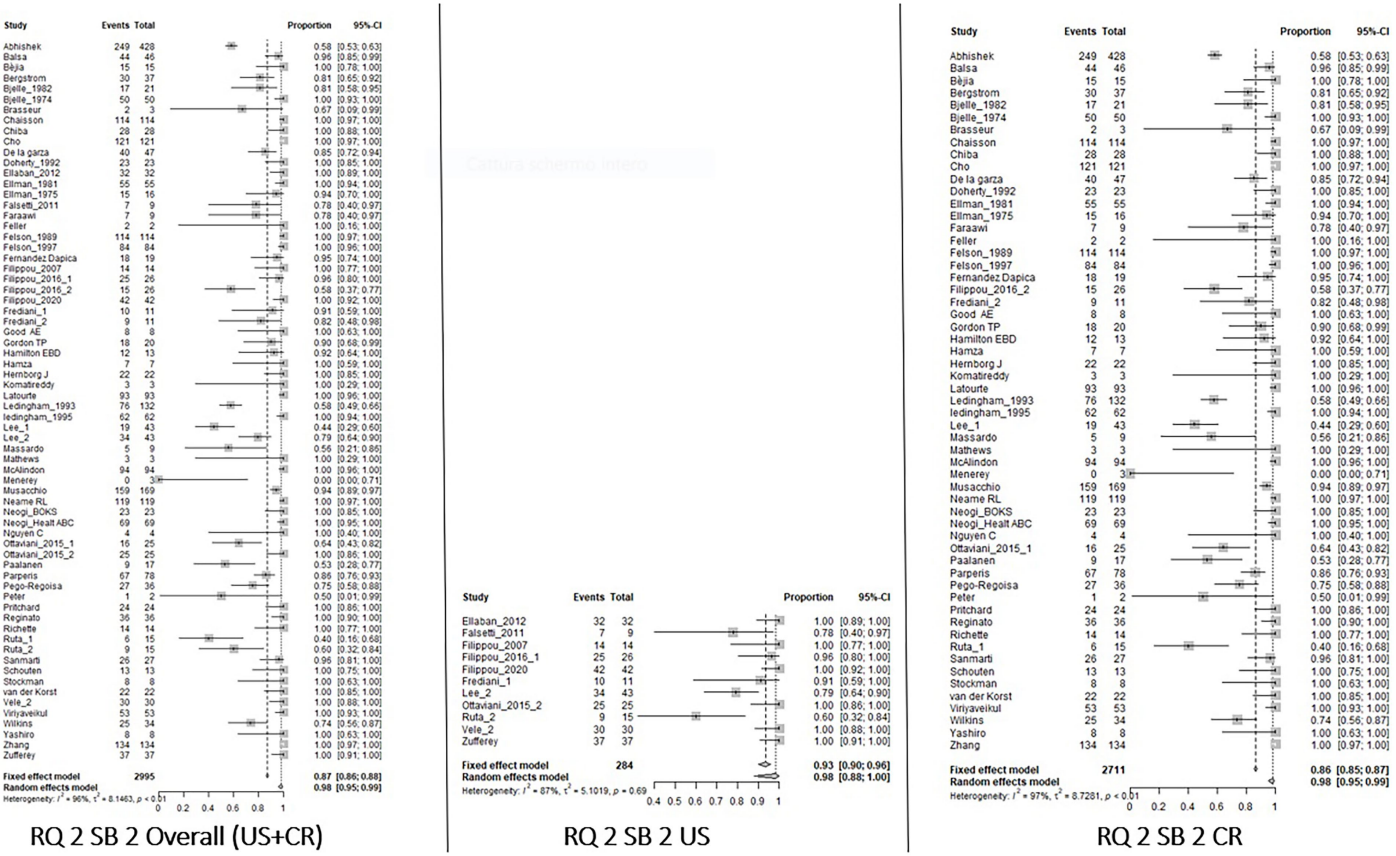
Forest Plot Knee, Research Question 2 (RQ 2), Sub_analysis 2: patients with suspected CPPD and knee/wrist used as index joint, independently of the reference standard used for diagnosis, analyzed by imaging.

**Figure 5:**
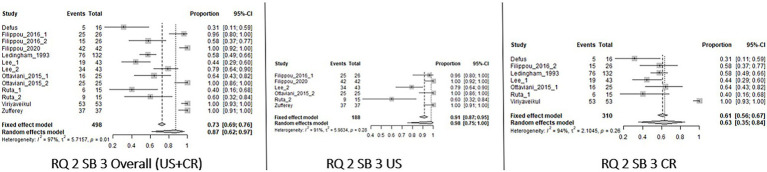
Forest Plot Knee, Research Question 2 (RQ 2), Sub-analysis 3: patients with suspected CPPD diagnosis and reference standard different than imaging, analyzed by imaging.

The results of the meta-analysis are summarized in Table 3. Forest Plots of the knees are shown in Figures 2–5. Forest plots of the other joints are shown in SP4.

**Table 3:** Meta-analyzes results.

Definite CPPD Diagnosis (RQ1)									
	Subanalysis 1			Subanalysis 2			Subanalysis 3		
	Prevalence (95% CI)			Prevalence (95% CI)			Prevalence (95% CI)		
Joint	Overall (US+CR)	US	CR	Overall (US+CR)	US	CR	Overall (US+CR)	US	CR
Knee	0.85 [0.62–0.95] (18, 19, 25–27, 29, 33, 35, 37–39)	0.93 [0.68–0.99] (18, 25–27)	0.79 [0.44–0.95] (18, 19, 29, 33, 35, 37–39)	0.85 [0.62–0.95] (18, 19, 25–27, 29, 33, 35, 37–39)	0.93 [0.68–0.99] (18, 25–27)	0.79 [0.44–0.95] (18, 19, 29, 33, 35, 37–39)	NA	NA	NA
Wrist	0.72 [0.47–0.88] (19, 25, 33, 35, 37)	NA	0.64 [0.39–0.83] (19, 33, 35, 37)	0.87 [0.74–0.94] (19–21, 28, 32–37)	0.92 [0.87–0.96] (20, 21, 25, 28)	0.83 [0.61–0.94] (19–21, 32–37)	NA	NA	NA
Hand	0.13 [0.03–0.42] (25, 33, 35, 37)	NA	0.17 [0.00–0.93] (33, 35, 37)	0.10 [0.03–0.26] (25, 32, 33, 35, 37)	NA	0.11 [0.01–0.54] (32, 33, 35, 37)	NA	NA	NA
Elbow		NA			NA		NA	NA	NA
Shoulder	0.42 [0.12–0.79] (33, 35, 37)	NA	0.42 [0.12–0.79] (33, 35, 37)	0.42 [0.12–0.79] (33, 35, 37)	NA	0.42 [0.12–0.79] (33, 35, 37)	NA	NA	NA
AC	NA	NA	NA	NA	NA	NA	NA	NA	NA
Hip	NA	NA	NA	NA	NA	NA	NA	NA	NA
Ankle	0.21 [0.05–0.56] (25, 29, 33, 35, 37)	NA	0.13 [0.03–0.41] (29, 33, 35, 37)	0.21 [0.05–0.56] (25, 29, 33, 35, 37)	NA	0.13 [0.03–0.41] (29, 33, 35, 37)	NA	NA	NA
Foot	NA	NA	NA	NA	NA	NA	NA	NA	NA
Suspected CPPD diagnosis (RQ2)									
	Subanalysis 1			Subanalysis 2			Subanalysis 3		
	Prevalence (95% CI)			Prevalence (95% CI)			Prevalence (95% CI)		
	Overall (US ± CR)	US	CR	Overall (US ± CR)	US	CR	Overall (US ± CR)	US	CR
Knee	0.98 [0.96–0.99] (40, 42–51, 55, 56, 58–76, 79–85, 88–91, 93, 95–102, 105–110)	0.98 [0.88–1.00] (56, 60, 66–69, 81, 91, 99, 110)	0.98 [0.96–0.99] (40, 42–51, 55, 58, 59, 61–65, 67, 69–76, 79–85, 88–91, 93–102, 105–109)	0.98 [0.95–0.99] (34, 40, 42–51, 55, 56, 58–76, 79–85, 88–91, 93–102, 105–109)	0.98 [0.88–1.00] (56, 60, 66–69, 81, 91, 99, 110)	0.98 [0.95–0.99] (34, 40, 42–51, 55, 58, 59, 61–65, 67, 69–76, 79–85, 88–91, 93–102, 105–109)	0.87 [0.62–0.97] (52, 67, 68, 79, 81, 91, 99, 106, 110)	0.98 [0.75–1.00] (67, 68, 81, 91, 99, 110)	0.63 [0.35–0.84] (40, 50, 52, 61, 67, 72, 78)
Wrist	0.51 [0.41–0.61] (40, 42, 44–47, 51, 56, 59, 61, 69–73, 82, 83, 85, 93, 95–97, 100, 102, 105, 107, 108, 110)	0.33 [0.13–0.63] (56, 69, 110)	0.53 [0.43–0.64] (40, 42, 44–47, 51, 58, 61, 69–73, 82, 83, 85, 93, 95–97, 100, 102, 105, 107, 108)	0.56 [0.45–0.66] (34, 40, 42, 44–47, 51, 53, 56, 57, 59, 61, 69–73, 82, 83, 85, 93, 95–97, 100, 102–105, 107, 108, 110)	0.33 [0.13–0.63] (56, 69, 110)	0.58 [048–0.68] (34, 40, 42, 44–47, 51, 53, 57, 59, 61, 69–73, 82, 83, 85, 93, 95–97, 100, 102–105, 107, 108)	NA	NA	NA
Suspected CPPD diagnosis (RQ2)									
Hand	0.18 [0.11–0.29] (40, 44, 73, 97, 100, 105, 107, 110)	NA	0.21 [0.13–0.32] (40, 44, 73, 97, 100, 105, 107)	0.18 [0.11–0.29] (40, 44, 73, 97, 100, 105, 107, 110)	NA	0.21 [0.13–0.32] (40, 44, 73, 97, 100, 105, 107)	NA	NA	NA
Elbow	0.41 [0.10–0.81] (45, 70, 73, 97)	NA	0.41 [0.10–0.81] (45, 70, 73, 97)	0.41 [0.10–0.81] (45, 70, 73, 97)	NA	0.41 [0.10–0.81] (45, 70, 73, 97)	NA	NA	NA
Shoulder	0.37 [0.15–0.66] (42, 69, 70, 73, 85, 93, 97, 105)	NA		0.37 [0.15–0.66] (42, 69, 70, 73, 85, 93, 97, 105)	NA	0.38 [0.14–0.70] (42, 69, 70, 73, 85, 93, 97, 105)	NA	NA	NA
AC	NA	NA	NA	NA	NA	NA	NA	NA	NA
Hip	0.27 [0.15–0.44] (40, 42, 45, 46, 59, 73, 85, 97, 102, 107)	NA	0.27 [0.15–0.44] (40, 42, 45, 46, 59, 73, 85, 97, 102, 107)	0.27 [0.15–0.44] (40, 42, 45, 46, 59, 73, 85, 97, 102, 107)	NA	0.27 [0.15–0.44] (40, 42, 45, 46, 73, 85, 97, 102, 107)	NA	NA	NA
Ankle	0.22 [0.05–0.60] (45, 73, 97, 110)	NA	0.34 [0.08–0.75] (45, 73, 97)	0.22 [0.05–0.60] (45, 73, 97, 110)	NA	0.34 [0.08–0.75] (45, 73, 97)	NA	NA	NA
Foot	0.36 [0.16–0.63] (73, 93, 97, 110)	NA	0.44 [0.17–0.74] (73, 93, 97)	0.36 [0.16–0.63] (73, 93, 97, 110)	NA	0.44 [0.17–0.74] (73, 93, 97)	NA	NA	NA

NA, Not Applicable; CI, Confidence Interval; US, Ultrasound; CR, Conventional Radiography; AC, Acromion-Clavicular.

### Assessment of the risk of bias

Most studies (88%, 83/94) showed a low risk of bias in many items, and the overall risk of bias was acceptable. Only a few studies had a high risk of bias (27, 53–55, 58, 65, 71, 90, 100, 104, 105).

Regarding the cohort study, the less fulfilled item was the comparability of the cohorts, which was related to the lack of matching of exposed and non-exposed patients or adjustment for confounders. Usually, the length of follow-up is not evaluable because most of the cohort studies included were mainly cross-sectional studies. For case–control studies, the main limitations were related to the representativeness of the cases and controls due to the lack of defined criteria for population selection. Finally, considering the diagnostic studies, the main source of bias was related to the reference standards used. In fact, in three studies, CR was applied as both index and reference standard (27, 53, 94), while in nine articles, the reference test was the McCarty criteria (20–24, 26, 29, 39, 60). All results of the NOS scale and QUADAS-2 are summarized in SP5.

## Discussion

Currently, assessing the prevalence of CPPD remains challenging, mainly because of the heterogeneity of its clinical manifestations (6) and the lack of a non-invasive and accurate diagnostic technique. Furthermore, the natural history of CPPD is still unclear, and the patterns of involvement of the peripheral joints in terms of extent and chronological order have not been defined. In fact, despite being the most evaluated knee joint in the literature, previous studies have shown that radiographic chondrocalcinosis is common in wrists and hips, even in the absence of knee involvement (20, 40).

These aspects make imaging a potential cornerstone for CPPD diagnosis and monitoring. In this scenario, US presents several advantages as a noninvasive examination that can be applied to many joints in a short time. Moreover, US has been validated by the OMERACT validation process for diagnosis (10, 111). To further improve the application of imaging in CPPD, identifying the joints most frequently affected would improve feasibility and accuracy.

The OMERACT Ultrasound Working group in CPPD performed this SLR to collect the available data on the prevalence of CPPD in peripheral joints, assessed both by US and/or CR, to identify the most relevant joints to scan for CPPD diagnosis and monitoring. In fact, this SLR is the first multi-step approach that will lead to the creation of an US scoring system for CPPD.

Unfortunately, among the included studies, several sources of heterogeneity emerged, as the articles varied in terms of the type and number of joints evaluated, reference standard used, index joint, and CPPD clinical features. These differences made the articles less comparable and introduced biases in the descriptive analysis. Assessing the studies included in RQ1 and RQ2, some differences may be appreciated: for RQ1 (definite diagnosis), imaging was mainly applied on a larger number of joints or on sites different than the knee (only three of 22 articles evaluated the knee alone), using the knee as index joint for the diagnosis, while in the RQ2 (suspected CPPD) the knee was the only joint assessed for diagnosis in almost 50% of the articles, reducing the number of other joints available for analysis. Specific meta-analyzes were performed to address these issues. For each research question, studies were divided according to the index joint for CPPD (knee or wrist) and the reference standard used for diagnosis (selecting only studies that included SFA or histology). This selection led to the identification of the most homogenous study groups, comparable in meta-analyzes that assessed the prevalence of CPP deposits but, on the other hand, reduced the number of patients included in the analysis.

Considering the descriptive analysis, the knee and wrist resulted in the joints being mostly involved in CPPD at both CR and US, independent of the research question. The CPPD prevalence was higher with US at both sites for RQ1, while in RQ2 a higher CPPD prevalence in the knee was detected by US than by CR. In contrast, CR revealed more cases in the wrist than US. Meta-analyzes supported these findings. In fact, according to meta-analyzes, the knee is the joint characterized by the highest CPPD prevalence in RQ2, with values constantly equal to 0.98 in SB_1 and SB_2 (no differences according to the imaging technique used), whereas the prevalence decreased in RQ1 with a higher value in US than in CR [0.93 (0.68–0.99) and 0.79 (0.44–0.95), respectively]. The higher prevalence of CPPD among suspected patients was a surprising result, but is probably explained by the predominant assessment of the knee in RQ2, and by the simultaneous use of CR as an index and reference test in most of the studies included.

The higher CPPD prevalence at the level of the knee when US was applied was also shown in SB_3, which assessed only articles with a reference standard different from imaging [prevalence values: US 0.98 (0–75-1.00), CR 0.63 (0.35–0.84)]. The higher CPPD prevalence by US could be due to the higher sensitivity of this technique compared to CR in detecting CPP deposits at the knee level, as shown in previous studies (11, 67).

The results of this SLR also confirmed the common involvement of the wrist in CPPD, even higher than the knee in RQ1 according to the meta-analysis [US 0.92 (0.87–0.96), CR 0.83 (0.61–0.94)] but not in RQ2 [US 0.33 (0.13–0.63), CR 0.58 (0.48–0.68)]. Surprisingly, CPPD prevalence in the wrist was higher in US only in RQ1 and not in RQ2, but this is probably due to the widespread use of CR in RQ2 studies. In fact, the limited data available in the literature regarding a comparative assessment of the wrist showed a higher capability of US in detecting CPP deposits (11). For other joints, the hip showed a lower prevalence than the elbow or shoulder (0.27, 0.41 and 0.37 in the hip, elbow, and shoulder, respectively), but these results were obtained in a small number of patients and should be further addressed. Furthermore, all results were obtained only by CR and could be different if US was applied. Finally, the hand was the joint characterized by the lowest CPPD prevalence, from the 0.10 at the RQ1 to the 0.18 of the RQ2.

In addition, the descriptive results of the articular structures substantially confirmed the prevalence distribution of the whole joint, with higher values at the menisci, TFC, and knee hyaline cartilage. Again, these results were mainly obtained through CR, which could have some limitations.

The results of this SLR, although interesting, should be carefully evaluated. The included studies were heterogeneous in design, population, and reference standards. However, some degree of heterogeneity could be expected, as this is a frequent finding in meta-analysis (112). A common limitation of these studies was the use of CR as an index and reference test simultaneously, leading to a potential misidentification of the CPPD given the low sensitivity of CR, and mainly to an overestimation of the prevalence of deposition. Another issue regarding the joints assessed is that most of the articles evaluated only the knee and/or wrist, and very little data were available for other joints, making the results poorly reliable. Finally, other potentially very sensitive imaging techniques, such as computed tomography (CT) or dual-energy CT, were not included in this systematic review because very little data were available.

On the other hand, this SLR was the first attempt to collect literature data about the distribution of CPP deposits at peripheral joints using imaging techniques mainly applied in clinical practice. Furthermore, this SLR provided results regarding the single joint’s structure and bilateral involvement, and these data could be useful in clinical practice. The strengths of this SLR were the identification of sub-groups, ability to reduce the sources of heterogeneity, and the inclusion of meta-analysis aimed to assess the impact of the factors that mainly affected CPPD recognition by imaging: the index joint used to identify the deposits, and the reference standard used to confirm it. Finally, the overall quality of the studies included in the present SLR was acceptable, and the risk of bias was low to moderate.

Considering all the issues that have emerged, the future research agenda should include studies providing polyarticular assessment of CPPD patients, the definition of a tool for monitoring CPPD, and the planning of prospective studies.

In conclusion, the results of this SLR showed that the knee and wrist have the highest CPPD prevalence and should be incorporated into the set of joints for a CPPD follow-up. Furthermore, a higher prevalence of CPP deposits in the US was confirmed. Further, this SLR highlighted the widespread heterogeneity of the studies on CPPD, especially regarding the reference standard applied. This SLR will be the starting point for the development of a US scoring system by the OMERACT US working group for CPPD that could place US as the most validated tool for CPPD assessment both in clinical practice and for research.

## Data availability statement

The original contributions presented in the study are included in the article/Supplementary material, further inquiries can be directed to the corresponding author.

## Author contributions

GF, GS, and CS contributed to the conception and design of the study. AA, EC, EF, FP, and SS collected the data. GS and AA organized the database. AZ and NU performed the statistical analysis. AA wrote the first draft of the manuscript. GS, GF, EF, and AZ wrote sections of the manuscript. All authors contributed to the manuscript revision, read, and approved the submitted version.

## Funding

This work was supported and funded by the Italian Ministry of Health: Ricerca Corrente.

## Conflict of interest

The authors declare that the research was conducted in the absence of any commercial or financial relationships that could be construed as a potential conflict of interest.

## Publisher’s note

All claims expressed in this article are solely those of the authors and do not necessarily represent those of their affiliated organizations, or those of the publisher, the editors and the reviewers. Any product that may be evaluated in this article, or claim that may be made by its manufacturer, is not guaranteed or endorsed by the publisher.

## Supplementary material

The Supplementary material for this article can be found online at:

https://www.frontiersin.org/articles/10.3389/fmed.2023.1131362/full#supplementary-material
