# Supplementary material for: Which are the most frequently involved peripheral joints in calcium pyrophosphate crystal deposition at imaging? A systematic literature review and meta-analysis by the OMERACT ultrasound – CPPD subgroup
Source: Front Med (Lausanne). 2023 Mar 9;10:1131362. doi: 10.3389/fmed.2023.1131362 (PMC10034772; doi:10.3389/fmed.2023.1131362)
Supplement: Supplementary file 1 [file Data_Sheet_1.pdf]

PubMed

1 Chondrocalcinosis[Mesh]

2 Chondrocalcinosis

3 Calcium pyrophosphate

4 Pseudogout

5 Calcium pyrophosphate dihydrate deposition

6 Calcium pyrophosphate dihydrate deposition disease

7 Calcium Gout

8 Calcium pyrophosphate arthropathy

9 OR 1-8

10 Ultrasonography[Mesh]

11 Ultrason*[All fields]

12 "Ultrasound"[all fields]

13 Sonograph*[all fields]

14 Ecograph*[all fields]

15 Echotomograph*[all fields]

16 ultrasonograph*[all fields]

17 OR 10-15

18 Tomography, X-Ray Computed[Mesh]

19 Contrast media[Mesh]

20 Cat scan*[all fields]

21 Ct[all fields]

22 OR 18-21

23 Radiography[Mesh]

24 X-Rays[Mesh]

25 Xray*[all fields]

26 Roentgen*[all fields]

27 Radiogram*[all fields]

28 OR 23-27

29 17 OR 22 OR 28

30 9 AND 29

Limits: Humans, Adults

Embase

1 Pseudogout/exp OR Pseudogout

2 Chondrocalcinosis/exp OR Chondrocalcinosis

3 Calcium pyrophosphate

4 OR 1-3

5 'radiography'/exp OR 'radiography'

6 radiogram*

7 roentgen*

8 radiograph*

9 OR 5-8

10 'ct scan'

11 'computer assisted tomography'/exp OR 'computer assisted tomography'

12 OR 10-11

13 'echography'/exp OR 'echography'

14 ultrasonograph*

15 'ultrasound'

16 OR 13-15

17 9 OR 12 OR 16

18 4 AND 17

Limits: Humans, Adults, Embase

LAST SEARCH 09/04/2021
