# Supplementary material for: Which are the most frequently involved peripheral joints in calcium pyrophosphate crystal deposition at imaging? A systematic literature review and meta-analysis by the OMERACT ultrasound – CPPD subgroup
Source: Front Med (Lausanne). 2023 Mar 9;10:1131362. doi: 10.3389/fmed.2023.1131362 (PMC10034772; doi:10.3389/fmed.2023.1131362)
Supplement: Supplementary file 2 [file Data_Sheet_2.pdf]

WRIST

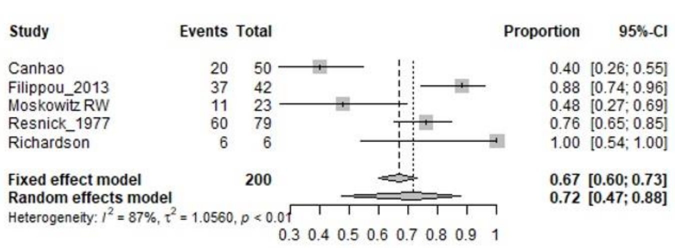

RQ 1 SB 1 Overall (US+CR)

RQ 1 SB 1 US

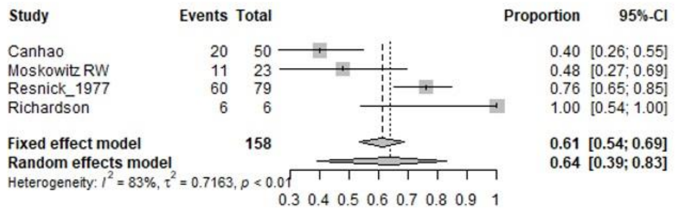

RQ 1 SB 1 CR

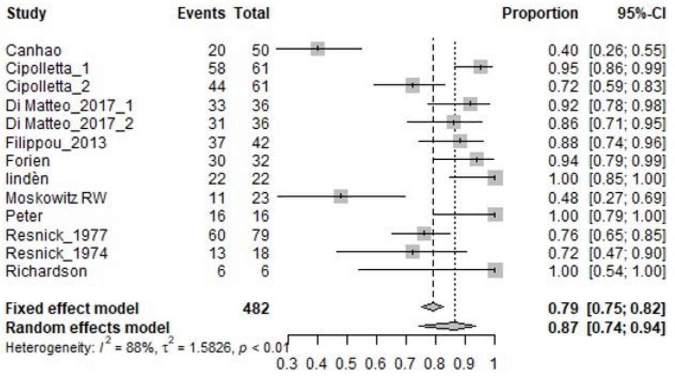

RQ 1 SB 2 Overall (US+CR)

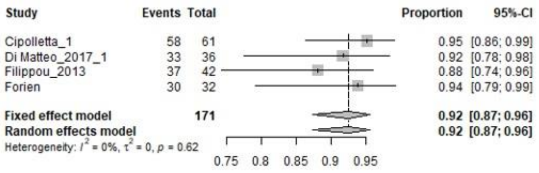

RQ 1 SB 2 US

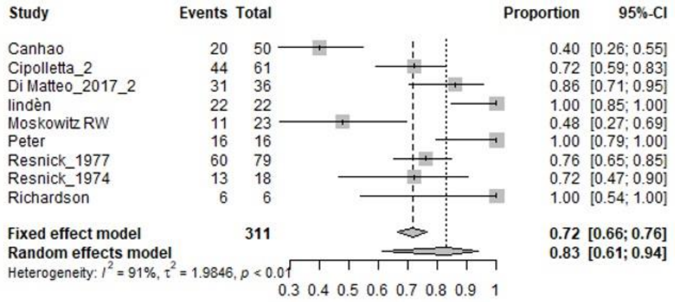

RQ 1 SB 2 CR

Forest Plot WRIST, Research question 1 (RQ1), Sub-analysis 1 and 2: patients with definite diagnosis of CPPD and knee as index joint (SB1) or knee and/or wrist (SB2) analysed by imaging

WRIST

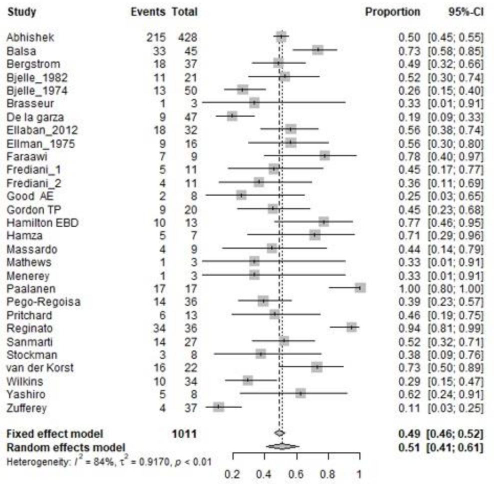

RQ 2 SB 1 Overall (US+CR)

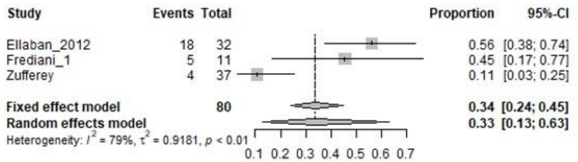

RQ 2 SB 1 US

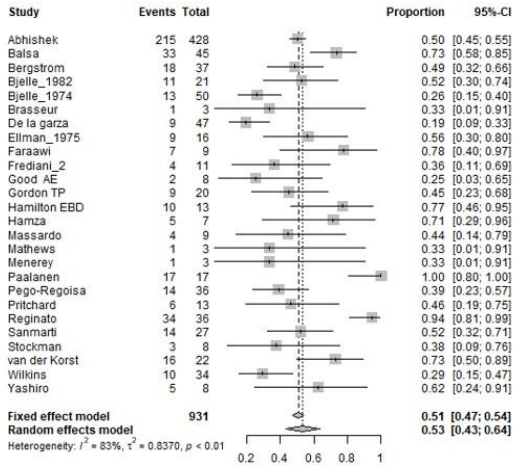

RQ 2 SB 1 CR

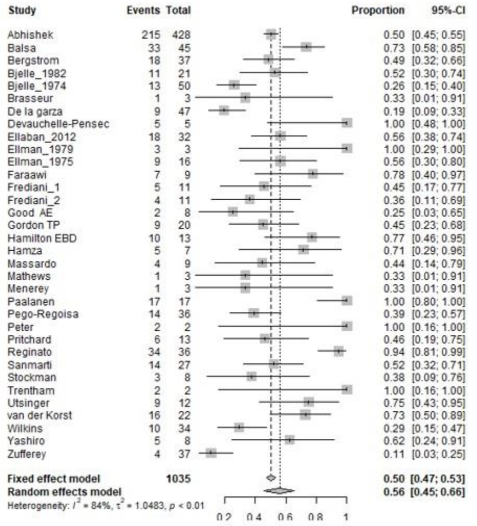

RQ 2 SB 2 Overall (US+CR)

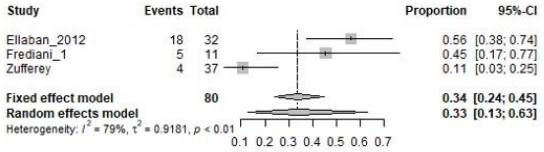

RQ 2 SB 2 US

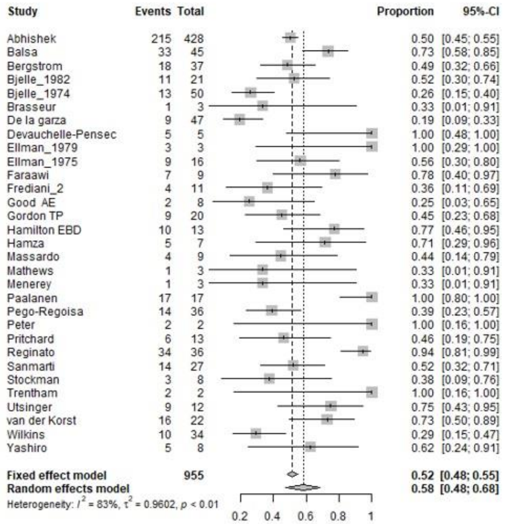

RQ 2 SB 2 CR

Forest Plot WRIST, Research question 2 (RQ2), Sub-analysis 1 and 2: patients with definite diagnosis of CPPD and knee as index joint (SB1) or knee and/or wrist (SB2) analysed by imaging

HAND

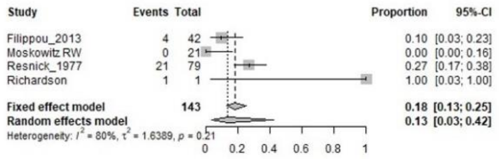

RQ 1 SB 1 Overall (US+CR)

RQ 1 SB 1 US

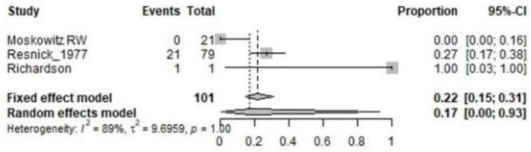

RQ 1 SB 1 CR

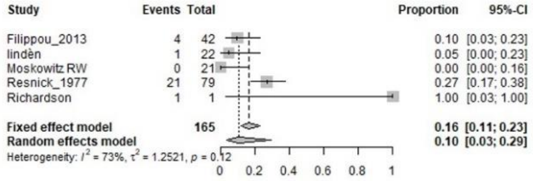

RQ 1 SB 2 Overall (US+CR)

RQ 1 SB 2 US

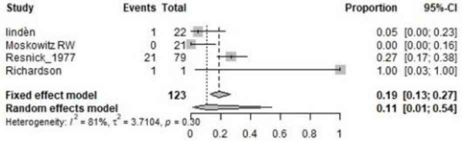

RQ 1 SB 2 CR

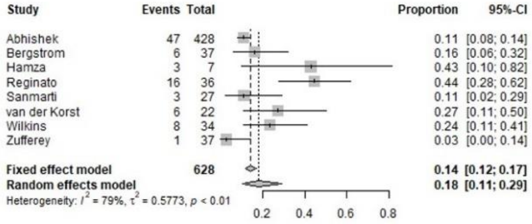

RQ 2 SB 1 Overall (US+CR)

RQ 2 SB 1 US

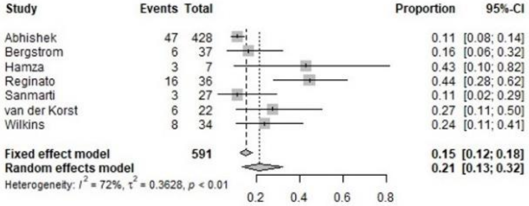

RQ 2 SB 1 CR

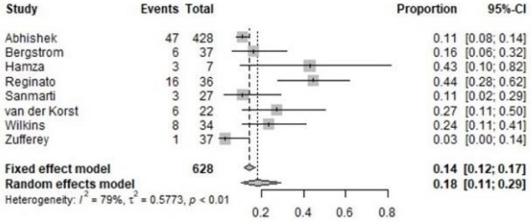

RQ 2 SB 2 Overall (US+CR)

RQ 2 SB 2 US

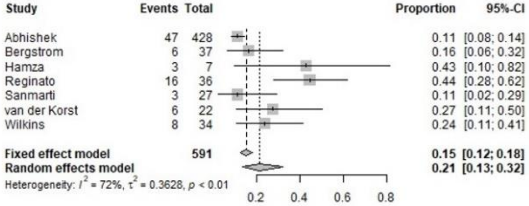

RQ 2 SB 2 CR

Forest Plot HAND, Research question 1 (RQ1) and 2 (RQ2), Sub-analysis 1 and 2: patients with definite diagnosis of CPPD and knee as index joint (SB1) or knee and/or wrist (SB2) analysed by imaging

SHOULDER

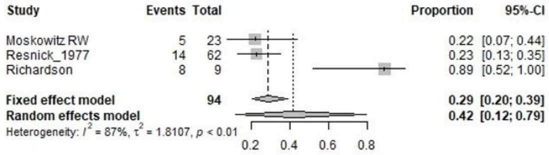

RQ 1 SB 1 Overall (US+CR)

RQ 1 SB 1 US

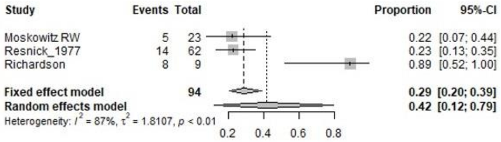

RQ 1 SB 1 CR

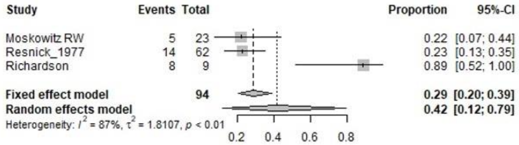

RQ 1 SB 2 Overall (US+CR)

RQ 1 SB 2 US

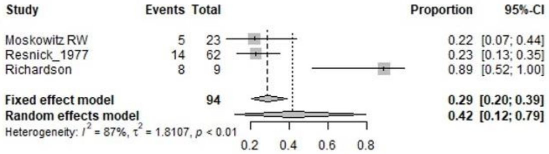

RQ 1 SB 2 CR

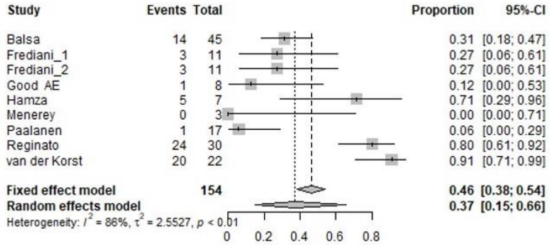

RQ 2 SB 1 Overall (US+CR)

RQ 2 SB 1 US

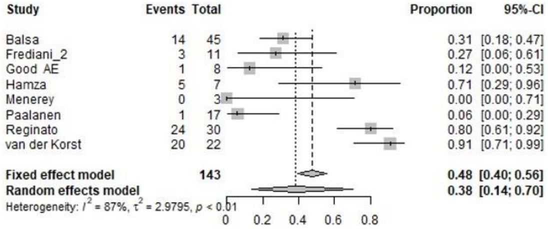

RQ 2 SB 1 CR

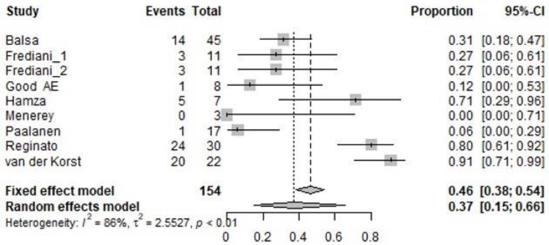

RQ 2 SB 2 Overall (US+CR)

RQ 2 SB 2 US

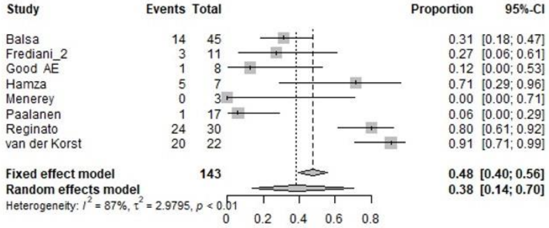

RQ 2 SB 2 CR

Forest Plot Shoulder, Research question 1 (RQ1) and 2 (RQ2), Sub-analysis 1 and 2: patients with definite diagnosis of CPPD and knee as index joint (SB1) or knee and/or wrist (SB2) analysed by imaging

ELBOW

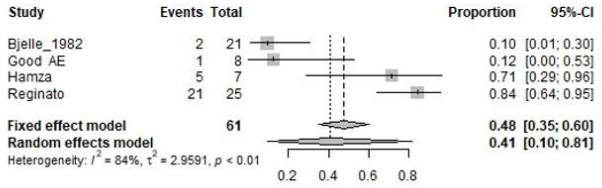

RQ 2 SB 1 Overall (US+CR)

RQ 2 SB 1 US

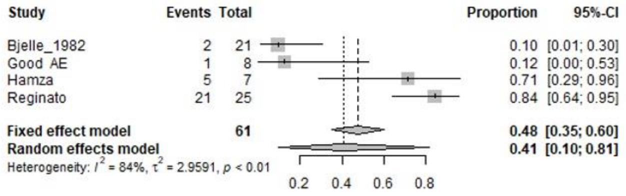

RQ 2 SB 1 CR

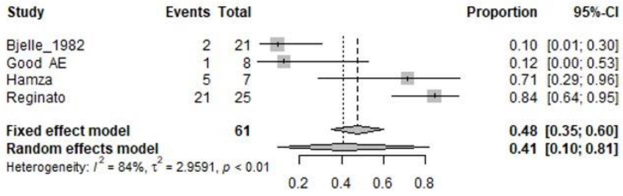

RQ 2 SB 2 Overall (US+CR)

RQ 2 SB 2 US

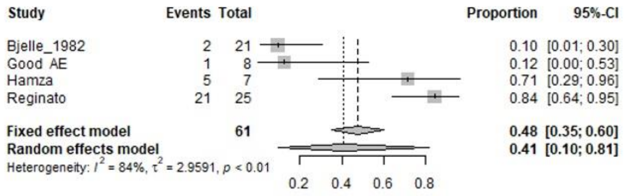

RQ 2 SB 2 CR

Forest Plot ELBOW, Research question 2 (RQ2), Sub-analysis 1 and 2: patients with definite diagnosis of CPPD and knee as index joint (SB1) or knee and/or wrist (SB2) analysed by imaging

HIP

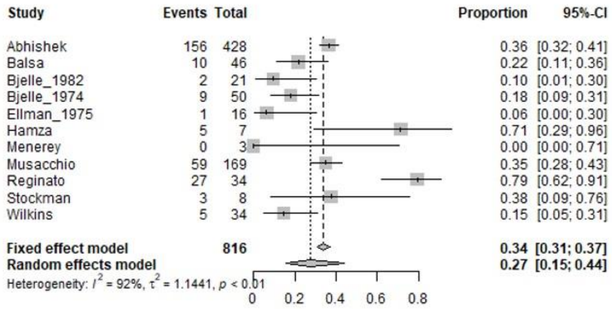

RQ 2 SB 1 Overall (US+CR)

RQ 2 SB 1 US

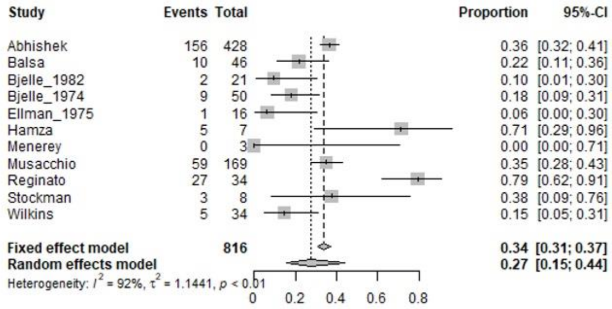

RQ 2 SB 1 CR

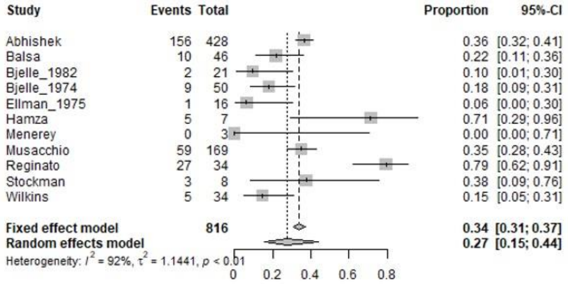

RQ 2 SB 2 Overall (US+CR)

RQ 2 SB 2 US

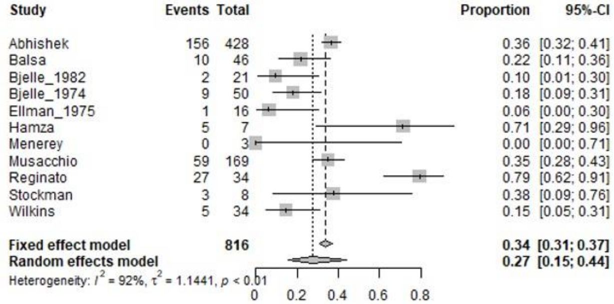

RQ 2 SB 2 CR

Forest Plot HIP, Research question 2 (RQ2), Sub-analysis 1 and 2: patients with definite diagnosis of CPPD and knee as index joint (SB1) or knee and/or wrist (SB2) analysed by imaging

ANKLE

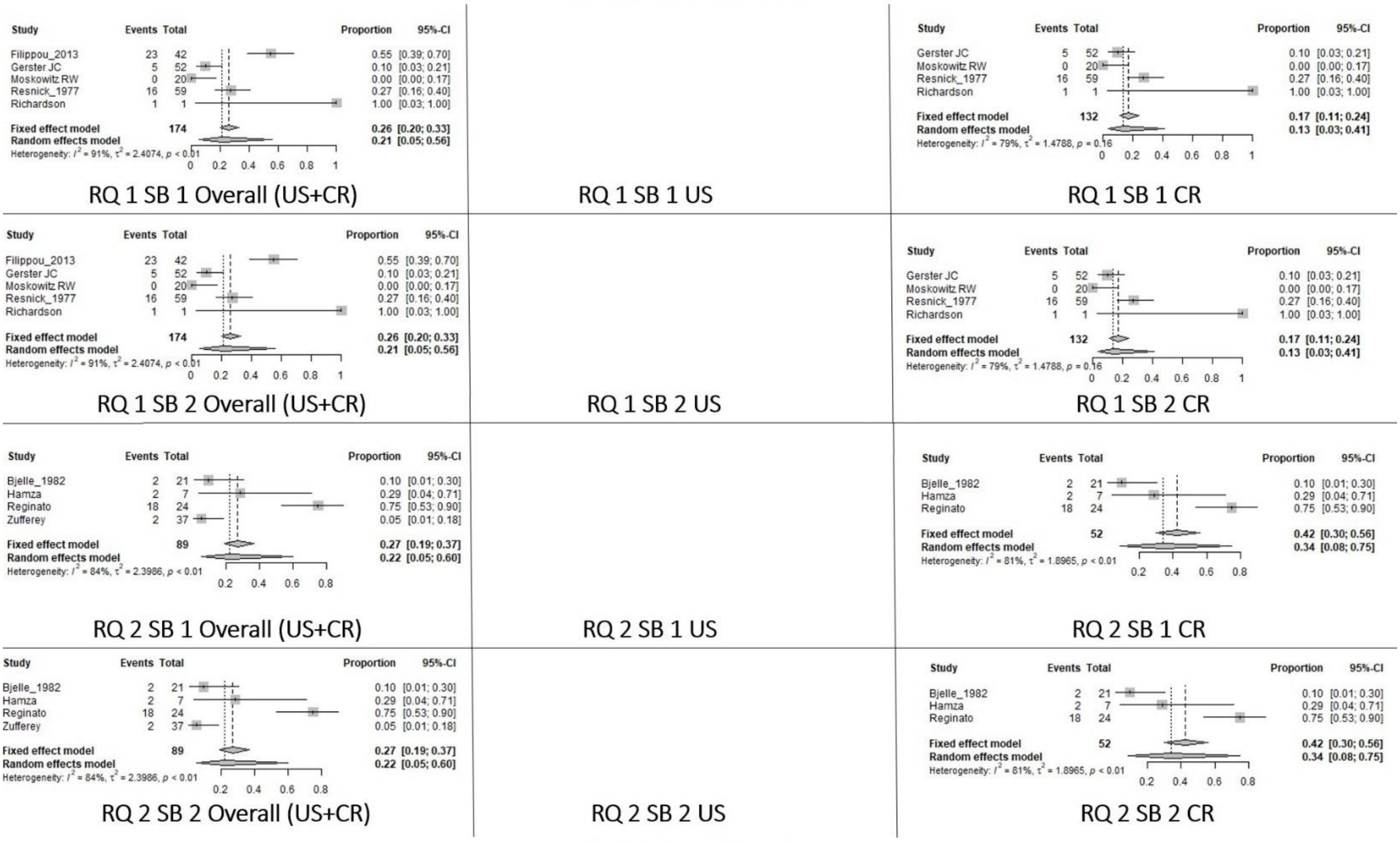

FOOT

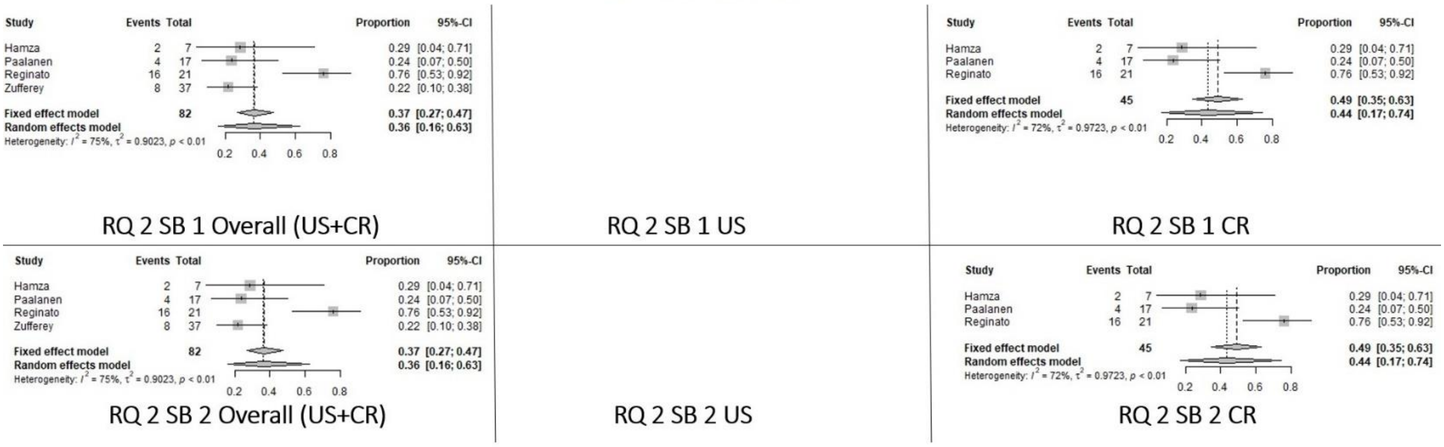

Forest Plot ANKLE, Research question 1 (RQ1) and 2 (RQ2), Sub-analysis 1 and 2: patients with definite diagnosis of CPPD and knee as index joint (SB1) or knee and/or wrist (SB2) analysed by imaging.

Forest Plot FOOT, Research Question 2 (RQ2), Sub-analysis 1 and 2: patients with definite diagnosis of CPPD and knee as index joint (SB1) or knee and/or wrist (SB2) analysed by imaging
