# Supplementary material for: Which are the most frequently involved peripheral joints in calcium pyrophosphate crystal deposition at imaging? A systematic literature review and meta-analysis by the OMERACT ultrasound – CPPD subgroup
Source: Front Med (Lausanne). 2023 Mar 9;10:1131362. doi: 10.3389/fmed.2023.1131362 (PMC10034772; doi:10.3389/fmed.2023.1131362)
Supplement: Supplementary file 3 [file Table_1.pdf]

Article	Joint	Imaging	CA_Mono Overall N	CA_Mono Pos N	CA Bil Overall N	CA Bilat Pos N	CA Bil N Pos Bilaterally	CA_UNK Overall N	CA_UNK Pos N	CO_Mono Overall N	CO_Mono Pos N	CO Bil Overall N	CO Bilat Pos N	CO Bil N pos bilaterally	CO_UNK Overall N	CO_UNK Pos N
Definite CPPD Diagnosis (RQ 1)																
Barskova et al, 2013	Knee	CR	NA	NA	25	13	6	NA	NA	NA	NA	0	NA	NA	NA	NA
Barskova et al, 2013	Knee	US	NA	NA	25	25	19	NA	NA	NA	NA	0	NA	NA	NA	NA
Cipolletta et al, 2020	Wrist	US	NA	NA	NA	NA	NA	61	58	NA	NA	NA	NA	NA	39	6
Cipolletta et al, 2020	Wrist	CR	NA	NA	NA	NA	NA	61	44	NA	NA	NA	NA	NA	39	0
Di Matteo et al, 2017	Wrist	US	NA	NA	36	33	23	NA	NA	NA	NA	48	9	0	NA	NA
Di Matteo et al, 2017	Wrist	CR	NA	NA	36	31	27	NA	NA	NA	NA	48	3	0	NA	NA
Di Matteo et al, 2019	Hip	US	NA	NA	NA	NA	NA	50	45	NA	NA	40	6	2	NA	NA
Di Matteo et al, 2019	Hip	XR	NA	NA	50	43	29	NA	NA	NA	NA	4’	4	1	NA	NA
Ellabban et al, 2012	Ankle	US	NA	NA	NA	NA	NA	38	22	NA	NA	NA	NA	NA	22	0
Falsetti et al, 2004	Ankle	US	NA	NA	57	33	25	NA	NA	NA	NA	100	0	NA	NA	NA
Falsetti et al, 2004	Ankle	XR	NA	NA	57	30	NA	NA	NA	NA	NA	100	0	NA	NA	NA
Filippou et al, 2013	Knee	US	NA	NA	42	41	36	NA	NA	NA	NA	NA	NA	NA	NA	NA
Filippou et al, 2013	Wrist	US	NA	NA	42	37	26	NA	NA	NA	NA	NA	NA	NA	NA	NA
Filippou et al, 2013	Hand	US	NA	NA	42	4	1	NA	NA	NA	NA	NA	NA	NA	NA	NA
Filippou et al, 2013	Ankle	US	NA	NA	42	11	5	NA	NA	NA	NA	NA	NA	NA	NA	NA
Filippucci et al, 2009	Knee	US	NA	NA	NA	NA	NA	48	33	NA	NA	NA	NA	NA	84	2

Forien et al, 2017	Wrist	US	NA	NA	NA	NA	NA	32	30	NA	NA	NA	NA	NA	26	4
Forien et al, 2017	Wrist	XR	NA	NA	NA	NA	NA	32	17	NA	NA	NA	NA	NA	26	0
Gerster et al, 1977	Knee	XR	NA	NA	52	5	2	NA	NA	NA	NA	52	0	NA	NA	NA
Gerster et al, 1977	Ankle	XR	NA	NA	52	5	2	NA	NA	NA	NA	52	0	NA	NA	NA
Huang et ak, 1993	Shoulder	XR	NA	NA	NA	NA	NA	17	7	NA	NA	NA	NA	NA	36	0
Huang et ak, 1993	AC	XR	NA	NA	17	17	9	NA	NA	NA	NA	NA	NA	NA	36	0
Foldes, 2002	Knee	US	21	17	NA	NA	NA	NA	NA	19	17	NA	NA	NA	NA	NA
Lindén et al, 1977	Wrist	XR	NA	NA	22	22	3	NA	NA	NA	NA	NA	NA	NA	NA	NA
Lindén et al, 1977	Hand	XR	NA	NA	22	1	0	NA	NA	NA	NA	NA	NA	NA	NA	NA
Moskowitz et al, 1967	Knee	XR	NA	NA	23	22	17	NA	NA	NA	NA	NA	NA	NA	NA	NA
Moskowitz et al, 1967	Wrist	XR	NA	NA	23	11	6	NA	NA	NA	NA	NA	NA	NA	NA	NA
Moskowitz et al, 1967	Hand	XR	NA	NA	21	0		NA	NA	NA	NA	NA	NA	NA	NA	NA
Moskowitz et al, 1967	Shoulder	XR	NA	NA	23	5	0	NA	NA	NA	NA	NA	NA	NA	NA	NA
Moskowitz et al, 1967	Hip	XR	NA	NA	23	7	3	NA	NA	NA	NA	NA	NA	NA	NA	NA
Moskowitz et al, 1967	Ankle	XR	NA	NA	20	0	NA	NA	NA	NA	NA	NA	NA	NA	NA	NA
Peter et al, 2001	Wrist	XR	NA	NA	NA	NA	NA	16	16	NA	NA	NA	NA	NA	NA	NA
Resnick et al, 1974	Wrist	XR	NA	NA	NA	NA	NA	18	13	NA	NA	NA	NA	NA	NA	NA

Resnick et al, 1977	Knee	XR	NA	NA	NA	NA	NA	80	68	NA	NA	NA	NA	NA	NA	NA
Resnick et al, 1977	Wrist	XR	NA	NA	NA	NA	NA	79	60	NA	NA	NA	NA	NA	NA	NA
Resnick et al, 1977	Hand	XR	NA	NA	NA	NA	NA	79	21	NA	NA	NA	NA	NA	NA	NA
Resnick et al, 1977	Elbow	XR	NA	NA	NA	NA	NA	56	33	NA	NA	NA	NA	NA	NA	NA
Resnick et al, 1977	Shoulder	XR	NA	NA	NA	NA	NA	62	14	NA	NA	NA	NA	NA	NA	NA
Resnick et al, 1977	AC	XR	NA	NA	NA	NA	NA	62	16	NA	NA	NA	NA	NA	NA	NA
Resnick et al, 1977	HIp	XR	NA	NA	NA	NA	NA	75	36	NA	NA	NA	NA	NA	NA	NA
Resnick et al, 1977	Ankle	XR	NA	NA	NA	NA	NA	59	16	NA	NA	NA	NA	NA	NA	NA
Resnick et al, 1977	Foot	XR	NA	NA	NA	NA	NA	58	14	NA	NA	NA	NA	NA	NA	NA
Richardson et al, 1983	Knee	XR	NA	NA	NA	NA	NA	11	10	NA	NA	NA	NA	NA	NA	NA
Richardson et al, 1983	Wrist	XR	NA	NA	NA	NA	NA	6	6	NA	NA	NA	NA	NA	NA	NA
Richardson et al, 1983	Hand	XR	NA	NA	NA	NA	NA	1	1	NA	NA	NA	NA	NA	NA	NA
Richardson et al, 1983	Shoulder	XR	NA	NA	NA	NA	NA	9	8	NA	NA	NA	NA	NA	NA	NA
Richardson et al, 1983	Ankle	XR	NA	NA	NA	NA	NA	1	1	NA	NA	NA	NA	NA	NA	NA
Richardson et al, 1983	Foot	XR	NA	NA	NA	NA	NA	1	1	NA	NA	NA	NA	NA	NA	NA
Schlesinger et al, 2009	Knee	XR	NA	NA	NA	NA	NA	67	20	NA	NA	NA	NA	NA	NA	NA
Vele et al, 2018	Knee	XR	NA	NA	30	30	16	NA	NA	NA	NA	NA	NA	NA	NA	NA

Vele et al, 2018	Knee	US	NA	NA	30	30	28	NA	NA	NA	NA	NA	NA	NA	NA	NA
Vele et al, 2018	Wrist	US	NA	NA	30	10	7	NA	NA	NA	NA	NA	NA	NA	NA	NA
Vele et al, 2018	Shoulder	US	NA	NA	30	0	NA	NA	NA	NA	NA	NA	NA	NA	NA	NA
Vele et al, 2018	AC	US	NA	NA	30	4	3	NA	NA	NA	NA	NA	NA	NA	NA	NA
Vele et al, 2018	Hip	US	NA	NA	30	2	1	NA	NA	NA	NA	NA	NA	NA	NA	NA
Vele et al, 2018	Ankle	US	NA	NA	30	1	1	NA	NA	NA	NA	NA	NA	NA	NA	NA
Suspected CPPD Diagnosis (RQ 2)																
Abhishek et al, 2012	Knee	CR	NA	NA	428	249	146	NA	NA	NA	NA	2690	0	0	NA	NA
Abhishek et al, 2012	Wrist	CR	NA	NA	428	215	134	NA	NA	NA	NA	2690	0	0	NA	NA
Abhishek et al, 2012	Hand	CR	NA	NA	428	47	34	NA	NA	NA	NA	2690	0	0	NA	NA
Abhishek et al, 2012	Hip	CR	NA	NA	428	156	62	NA	NA	NA	NA	2690	0	0	NA	NA
Ariyoshi et al, 2007	Knee	CR	NA	NA	NA	NA	NA	1	1	NA	NA	NA	NA	NA	0	0
Axford et al, 1991	Hip	CR	NA	NA	NA	NA	NA	17	17	NA	NA	NA	NA	NA	95	0
Balsa et al, 1990	Knee	CR	NA	NA	NA	NA	NA	46	44	NA	NA	NA	NA	NA	129	0
Balsa et al, 1990	Wrist	CR	NA	NA	NA	NA	NA	45	33	NA	NA	NA	NA	NA	129	0
Balsa et al, 1990	Shoulder	CR	NA	NA	NA	NA	NA	45	14	NA	NA	NA	NA	NA	129	0
Balsa et al, 1990	Hip	CR	NA	NA	NA	NA	NA	46	10	NA	NA	NA	NA	NA	129	0

Béija et al, 2004	Knee	CR	NA	NA	15	15	15	NA	NA	NA	NA	88	0	0	NA	NA
Bergstrom et al, 1986	Knee	CR	NA	NA	NA	NA	NA	37	30	NA	NA	NA	NA	NA	315	0
Bergstrom et al, 1986	Wrist	CR	NA	NA	NA	NA	NA	37	18	NA	NA	NA	NA	NA	315	0
Bergstrom et al, 1986	Hand	CR	NA	NA	NA	NA	NA	37	6	NA	NA	NA	NA	NA	315	0
Bjelle et al, 1982	Knee	CR	NA	NA	NA	NA	NA	21	17	NA	NA	NA	NA	NA	9	0
Bjelle et al, 1982	Wrist	CR	NA	NA	NA	NA	NA	21	11	NA	NA	NA	NA	NA	9	0
Bjelle et al, 1982	Elbow	CR	NA	NA	NA	NA	NA	21	2	NA	NA	NA	NA	NA	9	0
Bjelle et al, 1982	Shoulder	CR	NA	NA	NA	NA	NA	21	4	NA	NA	NA	NA	NA	9	0
Bjelle et al, 1982	Hip	CR	NA	NA	NA	NA	NA	21	2	NA	NA	NA	NA	NA	9	0
Bjelle et al, 1982	Ankle	CR	NA	NA	NA	NA	NA	21	2	NA	NA	NA	NA	NA	9	0
Bjelle et al, 1974	Knee	CR	NA	NA	NA	NA	NA	50	50	NA	NA	NA	NA	NA	250	0
Bjelle et al, 1974	Wrist	CR	NA	NA	NA	NA	NA	50	13	NA	NA	NA	NA	NA	250	0
Bjelle et al, 1974	Hip	CR	NA	NA	NA	NA	NA	50	9	NA	NA	NA	NA	NA	250	0
Brasseur et al, 1987	Knee	CR	NA	NA	NA	NA	NA	3	2	NA	NA	NA	NA	NA	97	0
Brasseur et al, 1987	Wrist	CR	NA	NA	NA	NA	NA	3	1	NA	NA	NA	NA	NA	97	0
Chaisson et al, 1996	Knee	CR	NA	NA	114	114	NA	NA	NA	NA	NA	1302	0	0	NA	NA
Checa et al, 2011	Knee	US	NA	NA	1	1	1	NA	NA	NA	NA	0	0	0	NA	NA

Checa et al, 2011	Wrist	US	NA	NA	1	1	1	NA	NA	NA	NA	0	0	0	NA	NA
Chiba et al, 2018	Knee	CR	NA	NA	28	28	16	NA	NA	NA	NA	1250	0	0	NA	NA
Cho et al, 2018	Knee	CR	NA	NA	121	121	19	NA	NA	NA	NA	4422	0	0	NA	NA
De la Garza et al, 2019	Knee	CR	NA	NA	47	40	19	NA	NA	NA	NA	1555	0	0	NA	NA
De la Garza et al, 2019	Wrist	CR	NA	NA	47	9	4	NA	NA	NA	NA	1555	0	0	NA	NA
Defus et al, 2002	Knee	CR	NA	NA	NA	NA	NA	16	5	NA	NA	NA	NA	NA	37	0
Devauchelle-Pensec et al, 2006	Wrist	CR	NA	NA	5	5	0	NA	NA	NA	NA	253	0	0	NA	NA
Doherty et al, 1996	Knee	CR	NA	NA	NA	NA	NA	43	43	NA	NA	NA	NA	NA	92	0
Doherty et al, 1992	Knee	CR	NA	NA	23	23	4	NA	NA	NA	NA	177	0	0	NA	NA
Ellabban et al, 2012	Knee	US	NA	NA	NA	NA	NA	32	32	NA	NA	NA	NA	NA	28	0
Ellabban et al, 2012	Wrist	US	NA	NA	NA	NA	NA	32	18	NA	NA	NA	NA	NA	28	0
Ellman et al, 1979	Wrist	CR	NA	NA	NA	NA	NA	3	3	NA	NA	NA	NA	NA	79	0
Ellman et al, 1981	Knee	CR	NA	NA	NA	NA	NA	55	55	NA	NA	NA	NA	NA	519	0
Ellman et al, 1975	Knee	CR	NA	NA	16	15	13	NA	NA	NA	NA	42	0	0	NA	NA
Ellman et al, 1975	Wrist	CR	NA	NA	16	9	8	NA	NA	NA	NA	42	0	0	NA	NA
Falsetti et al, 2011	Knee	US	NA	NA	NA	NA	NA	9	7	NA	NA	NA	NA	NA	52	3
Falsetti et al, 2011	Ankle	US	NA	NA	NA	NA	NA	9	6	NA	NA	NA	NA	NA	52	5

Feller et al, 1972	Knee	CR	NA	NA	NA	NA	NA	2	2	NA	NA	NA	NA	NA	15	0
Faraawi et al, 1993	Knee	CR	NA	NA	NA	NA	NA	9	7	NA	NA	NA	NA	NA	16	0
Faraawi et al, 1993	Wrist	CR	NA	NA	NA	NA	NA	9	7	NA	NA	NA	NA	NA	16	0
Felson et al, 1989	Knee	CR	NA	NA	NA	NA	NA	114	114	NA	NA	NA	NA	NA	1288	0
Felson et al, 1997	Knee	CR	NA	NA	NA	NA	NA	84	84	NA	NA	NA	NA	NA	895	0
Fernandez Dapica et al, 1986	Knee	CR	NA	NA	NA	NA	NA	19	18	NA	NA	NA	NA	NA	130	0
Filippou et al, 2007	Knee	US	NA	NA	NA	NA	NA	14	14	NA	NA	NA	NA	NA	29	0
Filippou et al, 2016	Knee	CR	NA	NA	NA	NA	NA	20	15	NA	NA	NA	NA	NA	14	1
Filippou et al, 2016	Knee	US	25	26	NA	NA	NA	NA	NA	16	2	NA	NA	NA	NA	NA
Filippou et al, 2020	Knee	US	NA	NA	NA	NA	NA	42	42	NA	NA	NA	NA	NA	25	0
Frediani et al, 2005	Knee	CR	NA	NA	NA	NA	NA	11	9	NA	NA	NA	NA	NA	13	0
Frediani et al, 2005	Knee	US	NA	NA	NA	NA	NA	11	10	NA	NA	NA	NA	NA	13	0
Frediani et al, 2005	Wrist	CR	NA	NA	NA	NA	NA	11	4	NA	NA	NA	NA	NA	13	0
Frediani et al, 2005	Wrist	US	NA	NA	NA	NA	NA	11	5	NA	NA	NA	NA	NA	13	
Frediani et al, 2005	Shoulder	CR	NA	NA	NA	NA	NA	11	3	NA	NA	NA	NA	NA	13	0
Frediani et al, 2005	Shoulder	US	NA	NA	NA	NA	NA	11	3	NA	NA	NA	NA	NA	13	0
Good et al, 1967	Knee	CR	1	1	7	7	3	NA	NA	NA	NA	NA	NA	NA	73	0

Commented [aa1]: Manca come numero nei controlli

Commented [aa2]: Manca come numero nei casi

Good et al, 1967	Wrist	CR	NA	NA	NA	NA	NA	8	2	NA	NA	NA	NA	NA	73	0
Good et al, 1967	Elbow	CR	NA	NA	NA	NA	NA	8	1	NA	NA	NA	NA	NA	73	0
Good et al, 1967	Shoulder	CR	NA	NA	NA	NA	NA	8	1	NA	NA	NA	NA	NA	73	0
Gordon et al, 1984	Knee	CR	NA	NA	NA	NA	NA	20	18	NA	NA	NA	NA	NA	107	0
Gordon et al, 1984	Wrist	CR	NA	NA	NA	NA	NA	20	9	NA	NA	NA	NA	NA	107	0
Hamilton EBD et al, 1972	Knee	CR	NA	NA	NA	NA	NA	1	1	NA	NA	NA	NA	NA	0	0
Hamilton EBD et al, 1972	Wrist	CR	NA	NA	NA	NA	NA	1	1	NA	NA	NA	NA	NA	0	0
Hamilton EBD et al, 1972	Hand	CR	NA	NA	NA	NA	NA	1	1	NA	NA	NA	NA	NA	0	0
Hamilton EBD et al, 1972	Hip	CR	NA	NA	NA	NA	NA	1	1	NA	NA	NA	NA	NA	0	0
Hamilton EBD et al, 1972	Ankle	CR	NA	NA	NA	NA	NA	1	1	NA	NA	NA	NA	NA	0	0
Hamilton EBD et al, 1981	Knee	CR	NA	NA	NA	NA	NA	13	12	NA	NA	NA	NA	NA	5	0
Hamilton EBD et al, 1981	Wrist	CR	NA	NA	NA	NA	NA	13	10	NA	NA	NA	NA	NA	5	0
Hamilton EBD et al, 1981	Hip	CR	NA	NA	NA	NA	NA	13	5	NA	NA	NA	NA	NA	5	0
Hamza et al, 1989	Knee	CR	NA	NA	NA	NA	NA	1	1	NA	NA	NA	NA	NA	0	0
Hamza et al, 1989	Wrist	CR	NA	NA	NA	NA	NA	1	1	NA	NA	NA	NA	NA	0	0
Hamza et al, 1989	Elbow	CR	NA	NA	NA	NA	NA	1	1	NA	NA	NA	NA	NA	0	0
Hamza et al, 1989	Hip	CR	NA	NA	NA	NA	NA	1	1	NA	NA	NA	NA	NA	0	0

Hamza et al, 1992	Knee	CR	NA	NA	7	7	6	NA	NA	NA	NA	70	0	0	NA	NA
Hamza et al, 1992	Wrist	CR	NA	NA	7	5	4	NA	NA	NA	NA	70	0	0	NA	NA
Hamza et al, 1992	Hand	CR	NA	NA	7	3	2	NA	NA	NA	NA	70	0	0	NA	NA
Hamza et al, 1992	Elbow	CR	NA	NA	7	5	5	NA	NA	NA	NA	70	0	0	NA	NA
Hamza et al, 1992	Shoulder	CR	NA	NA	7	5	4	NA	NA	NA	NA	70	0	0	NA	NA
Hamza et al, 1992	Hip	CR	NA	NA	7	5	5	NA	NA	NA	NA	70	0	0	NA	NA
Hamza et al, 1992	Ankle	CR	NA	NA	7	2	2	NA	NA	NA	NA	70	0	0	NA	NA
Hamza et al, 1992	Foot	CR	NA	NA	7	2	2	NA	NA	NA	NA	70	0	0	NA	NA
Hernborg et al, 1977	Knee	CR	NA	NA	NA	NA	NA	22	22	NA	NA	NA	NA	NA	62	0
Komatireddy et al, 1989	Knee	CR	NA	NA	NA	NA	NA	3	3	NA	NA	NA	NA	NA	77	0
Latourte et al, 2020	Knee	CR	NA	NA	93	93	50	NA	NA	NA	NA	563	0	0	NA	NA
Ledingham et al, 1992	Hip	CR	NA	NA	NA	NA	NA	23	2	NA	NA	NA	NA	NA	188	0
Ledingham et al, 1993	Hip	CR	NA	NA	NA	NA	NA	13	13	NA	NA	NA	NA	NA	123	0
Ledingham et al, 1993	Knee	CR	NA	NA	NA	NA	NA	132	76	NA	NA	NA	NA	NA	120	0
Ledingham et al, 1995	Knee	CR	NA	NA	NA	NA	NA	62	62	NA	NA	NA	NA	NA	126	0
Lee et al, 2019	Knee	CR	NA	NA	NA	NA	NA	43	19	NA	NA	NA	NA	NA	131	4
Lee et al, 2019	Knee	US	NA	NA	NA	NA	NA	43	34	NA	NA	NA	NA	NA	131	23

Massardo et al, 1989	Knee	CR	NA	NA	NA	NA	NA	9	5	NA	NA	NA	NA	NA	22	0
Massardo et al, 1989	Wrist	CR	NA	NA	NA	NA	NA	9	4	NA	NA	NA	NA	NA	22	0
Mathews et al, 1987	Knee	CR	NA	NA	NA	NA	NA	3	3	NA	NA	NA	NA	NA	42	0
Mathews et al, 1987	Wrist	CR	NA	NA	NA	NA	NA	3	1	NA	NA	NA	NA	NA	42	0
McAlindon et al, 1996	Knee	CR	NA	NA	NA	NA	NA	94	94	NA	NA	NA	NA	NA	506	0
Menerey et al, 1988	Knee	CR	NA	NA	NA	NA	NA	3	0	NA	NA	NA	NA	NA	19	0
Menerey et al, 1988	Wrist	CR	NA	NA	NA	NA	NA	3	1	NA	NA	NA	NA	NA	19	0
Menerey et al, 1988	Shoulder	CR	NA	NA	NA	NA	NA	3	0	NA	NA	NA	NA	NA	19	0
Menerey et al, 1988	Hip	CR	NA	NA	NA	NA	NA	3	0	NA	NA	NA	NA	NA	19	0
Montgomery et al, 1998	Hip	CR	3	2	1	0	0	NA	NA	8	0	3	0	0	NA	NA
Musacchio et al, 2011	Knee	CR	NA	NA	169	159	114	NA	NA	NA	NA	1460	0	0	NA	NA
Musacchio et al, 2011	Hip	CR	NA	NA	NA	NA	NA	169	59	NA	NA	NA	NA	NA	1460	0
Neame et al, 2003	Knee	CR	NA	NA	NA	NA	NA	119	119	NA	NA	NA	NA	NA	1608	0
Neogi et al (BOKS) 2006*	Knee	CR	NA	NA	NA	NA	NA	23	23	NA	NA	NA	NA	NA	242	0
Neogi et al (HEALTH ABC), 2006*	Knee	CR	NA	NA	NA	NA	NA	69	69	NA	NA	NA	NA	NA	161	0
Nguyen et al, 2013	Knee	CR	NA	NA	NA	NA	NA	4	4	NA	NA	NA	NA	NA	16	0
Ottaviani et al, 2015	Knee	CR	NA	NA	NA	NA	NA	25	16	NA	NA	NA	NA	NA	26	0

Ottaviani et al, 2015	Knee	US	NA	NA	NA	NA	NA	25	25	NA	NA	NA	NA	NA	26	2
Ottaviani et al, 2020	AC	US	NA	NA	29	29	NA	NA	NA	NA	NA	46	0	0	NA	NA
Paalanen et al, 2020	Knee	CR	NA	NA	NA	NA	NA	17	9	NA	NA	NA	NA	NA	418	0
Paalanen et al, 2020	Wrist	CR	NA	NA	NA	NA	NA	17	17	NA	NA	NA	NA	NA	418	0
Paalanen et al, 2020	Shoulder	CR	NA	NA	NA	NA	NA	17	1	NA	NA	NA	NA	NA	418	0
Paalanen et al, 2020	Foot	CR	NA	NA	NA	NA	NA	17	4	NA	NA	NA	NA	NA	418	0
Parperis et al, 2013	Knee	CR	NA	NA	NA	NA	NA	67	67	NA	NA	NA	NA	NA	422	0
Parperis et al, 2013	AC	CR	NA	NA	NA	NA	NA	21	21	NA	NA	NA	NA	NA	1899	0
Pego-Reigosa et al, 2005	Knee	CR	NA	NA	NA	NA	NA	36	27	NA	NA	NA	NA	NA	82	2
Pego-Reigosa et al, 2005	Wrist	CR	NA	NA	NA	NA	NA	36	14	NA	NA	NA	NA	NA	82	0
Peter et al, 2001	Knee	CR	NA	NA	NA	NA	NA	2	1	NA	NA	NA	NA	NA	25	0
Peter et al, 2001	Wrist	CR	NA	NA	NA	NA	NA	2	2	NA	NA	NA	NA	NA	25	0
Pritchard et al, 1977	Knee	CR	NA	NA	24	24	23	NA	NA	NA	NA	117	0	0	NA	NA
Pritchard et al, 1977	Wrist	CR	NA	NA	13	6	6	NA	NA	NA	NA	128	0	0	NA	NA
Reginato et al, 1976	Knee	CR	NA	NA	36	36	36	NA	NA	NA	NA	172	0	0	NA	NA
Reginato et al, 1976	Wrist	CR	NA	NA	36	34	30	NA	NA	NA	NA	172	0	0	NA	NA
Reginato et al, 1976	Hand	CR	NA	NA	36	16	15	NA	NA	NA	NA	172	0	0	NA	NA

Reginato et al, 1976	Elbow	CR	NA	NA	30	24	21	NA	NA	NA	NA	178	0	0	NA	NA
Reginato et al, 1976	Shoulder	CR	NA	NA	36	36	36	NA	NA	NA	NA	172	0	0	NA	NA
Reginato et al, 1976	Hip	CR	NA	NA	34	27	25	NA	NA	NA	NA	174	0	0	NA	NA
Reginato et al, 1976	Ankle	CR	NA	NA	24	18	18	NA	NA	NA	NA	184	0	0	NA	NA
Reginato et al, 1976	Foot	CR	NA	NA	21	16	15	NA	NA	NA	NA	187	0	0	NA	NA
Richette et al, 2007	Knee	CR	NA	NA	NA	NA	NA	14	14	NA	NA	NA	NA	NA	130	0
Ruta et al, 2016	Knee	CR	15	6	NA	NA	NA	NA	NA	60	10	NA	NA	NA	NA	NA
Ruta et al, 2016	Knee	US	15	9	NA	NA	NA	NA	NA	60	2	NA	NA	NA	NA	NA
Sanmarti et al, 1993	Knee	CR	NA	NA	NA	NA	NA	27	26	NA	NA	NA	NA	NA	234	0
Sanmarti et al, 1993	Wrist	CR	NA	NA	NA	NA	NA	27	14	NA	NA	NA	NA	NA	234	0
Sanmarti et al, 1993	Hand	CR	NA	NA	NA	NA	NA	27	3	NA	NA	NA	NA	NA	234	0
Schouten et al, 1992	Knee	CR	NA	NA	NA	NA	NA	13	13	NA	NA	NA	NA	NA	129	0
Stockman et al, 1980	Knee	CR	NA	NA	8	8	5	NA	NA	NA	NA	272	0	0	NA	NA
Stockman et al, 1980	Wrist	CR	NA	NA	8	3	NA	NA	NA	NA	NA	272	0	0	NA	NA
Stockman et al, 1980	Hip	CR	NA	NA	8	3	NA	NA	NA	NA	NA	272	0	0	NA	NA
Trentham et al, 1975	Wrist	CR	NA	NA	NA	NA	NA	2	2	NA	NA	NA	NA	NA	98	0
Utsingeret al, 1975	Wrist	CR	NA	NA	12	9	NA	NA	NA	NA	NA	6	0	0	NA	NA

van der Korst et al, 1974	Knee	CR	NA	NA	22	22	22	NA	NA	NA	NA	86	0	0	NA	NA
van der Korst et al, 1974	Wrist	CR	NA	NA	22	16	12	NA	NA	NA	NA	86	0	0	NA	NA
van der Korst et al, 1974	Hand	CR	NA	NA	22	6	6	NA	NA	NA	NA	86	0	0	NA	NA
van der Korst et al, 1974	Shoulder	CR	NA	NA	22	20	18	NA	NA	NA	NA	86	0	0	NA	NA
Viriyavejkul et al, 2007	Knee	CR	53	53	NA	NA	NA	NA	NA	49	0	NA	NA	NA	NA	NA
Wilkins et al, 1983	Knee	CR	NA	NA	34	25	16	NA	NA	NA	NA	66	0	0	NA	NA
Wilkins et al, 1983	Wrist	CR	NA	NA	34	10	5	NA	NA	NA	NA	66	0	0	NA	NA
Wilkins et al, 1983	Hand	CR	NA	NA	34	8	3	NA	NA	NA	NA	66	0	0	NA	NA
Wilkins et al, 1983	Hip	CR	NA	NA	34	5	4	NA	NA	NA	NA	66	0	0	NA	NA
Yashiro et al, 1991	Knee	CR	NA	NA	NA	NA	NA	8	8	NA	NA	NA	NA	NA	124	0
Yashiro et al, 1991	Wrist	CR	NA	NA	NA	NA	NA	8	5	NA	NA	NA	NA	NA	124	0
Zhang et al, 2004	Knee	CR	NA	NA	134	134	NA	NA	NA	NA	NA	1709	0	0	NA	NA
Zufferey et al, 2015	Knee	US	NA	NA	NA	NA	NA	37	37	NA	NA	NA	NA	NA	72	33
Zufferey et al, 2015	Wrist	US	NA	NA	NA	NA	NA	37	4	NA	NA	NA	NA	NA	72	2
Zufferey et al, 2015	Hand	US	NA	NA	NA	NA	NA	37	1	NA	NA	NA	NA	NA	72	1
Zufferey et al, 2015	Ankle	US	NA	NA	NA	NA	NA	37	2	NA	NA	NA	NA	NA	72	5
Zufferey et al, 2015	Foot	US	NA	NA	NA	NA	NA	37	8	NA	NA	NA	NA	NA	72	11

CA-cases, CO-controls, Mono-Monolateral evaluation, Bilat- Bilateral evaluation, UNK- Unknown laterality, CR-conventional radiology, US- Ultrasound

N.B Gutierrez et al, 2014 not included due to missing data at joint level
