# Supplementary material for: Which are the most frequently involved peripheral joints in calcium pyrophosphate crystal deposition at imaging? A systematic literature review and meta-analysis by the OMERACT ultrasound – CPPD subgroup
Source: Front Med (Lausanne). 2023 Mar 9;10:1131362. doi: 10.3389/fmed.2023.1131362 (PMC10034772; doi:10.3389/fmed.2023.1131362)
Supplement: Supplementary file 4 [file Table_2.pdf]

Confirmed CPPD Diagnosis (RQ1)

	Joint	***KNEE***			***WRIST***			***HAND***			***ELBOW***		***SHOULDER***			***HIP***		***ANKLE***		***FOOT***	
	Structure	**FC**	**HC**	**T**	**FC**	**HC**	**T**	**FC**	**HC**	**T**	**HC**	**T**	**FC**	**HC**	**T**	**FC**	**HC**	**HC**	**T**	**HC**	**T**
**CR**	**Imaging positive cases/all cases**	172/292 59%	80/242 33%	8/189 4%	139/293 47%	50/85 59%	NA	NA	20/85 24%	5/85 6%	27/85 32%	9/85 11%	3/85 4%	9/85 11%	6/85 7%	32/85 38%	21/85 25%	16/85 19%	5/104 5%	9/85 11%	10/85 12%
	**Cases positive bilaterally**	NA	NA	2/5* 40%	27/31* 87%	NA	NA	NA	NA	NA	NA	NA	NA	NA	NA	NA	NA	NA	2/5 40%	NA	0
**US**	**Imaging positive cases/all cases**	67/74* 90%	94/143 66%	0	70/126 56%	NA	NA	0	4/42 10%	NA	NA	NA	NA	NA	0	45/90 50%	13/90 14%	0	62/259 24%	0	0
	**Cases positive bilaterally**	NA	NA	NA	47/70 67%	NA	NA	0	1/4 25%	NA	NA	NA	NA	NA	NA	NA	NA	NA	38/56 68%	0	0

Suspected CPPD Diagnosis (RQ2)

	Joint	***KNEE***			***WRIST***			***HAND***			***ELBOW***		***SHOULDER***			***HIP***		***ANKLE***		***FOOT***	
	**Structure**	**FC**	**HC**	**T**	**FC**	**HC**	**T**	**FC**	**HC**	**T**	**HC**	**T**	**FC**	**HC**	**T**	**FC**	**HC**	**HC**	**FC**	**HC**	**T**
**CR**	**Imaging positive cases/all cases**	266/2937 9%	137/2369 6%	10/118 8%	43/823 5%	6/100 6%	NA	NA	NA	NA	NA	NA	0	20/108 19%	0	2/100 2%	3/100 3%	NA	NA	NA	NA
	**Cases positive bilaterally**	80/289 28%	57/252 23%	NA	6/80 9%	3/34 8%	NA	NA	NA	NA	NA	NA	0	18/22 81%	0	2/34 6%	2/34 6%	NA	NA	NA	NA
**US**	**Imaging positive cases/all cases**	111/464 24%	59/403 15%	5/174 3%	18/60 30%	NA	NA	NA	NA	NA	NA	NA	NA	NA	NA	NA	NA	NA	NA	NA	NA
	**Cases positive bilaterally**	NA	NA	NA	NA	NA	NA	NA	NA	NA	NA	NA	NA	NA	NA	NA	NA	NA	NA	NA	NA

*evaluated in a single study

Legend: US- Ultrasound, CR- Conventional Radiography, NA Not Applicable, FC- Fibrocartilage, HC-Hyaline Cartilage, T-Tendon

RQ1- Research Question 1, RQ2- Research Question2
