# Supplementary material for: Which are the most frequently involved peripheral joints in calcium pyrophosphate crystal deposition at imaging? A systematic literature review and meta-analysis by the OMERACT ultrasound – CPPD subgroup
Source: Front Med (Lausanne). 2023 Mar 9;10:1131362. doi: 10.3389/fmed.2023.1131362 (PMC10034772; doi:10.3389/fmed.2023.1131362)
Supplement: Supplementary file 5 [file Table_3.pdf]

Newcastle Ottawa Scale (NOS)- Cohort studies

	Selection				*Comparability*	*Exposure*		
**Study**	Representativeness of the exposed cohort	Selection of the non exposed cohort	Ascertainment of exposure	Demonstration that outcome of interest was not present at start of study	Comparability of cohorts	Assessment of outcome	Was follow-up long enough for outcomes to occurr	Adequacy of follow-up of cohorts
Abhishek 2012	*	*	*	*		*		
Axford 1991	*		*	*		*		*
Balsa 1990	*	*	*	*	*	*		*
Bergstrom 1986	*	*	*	*		*	*	*
Bjelle 1974	*		*	*		*		*
Canhão 2001	*		*	*		*		*
Chiba 2018	*	*	*	*		*		*
Cho 2018	*	*	*	*		*		*
De La Garza 2019			*	*	*	*		*
Derfus 2002	*	*	*	*		*		*
Faraawi 1993	*		*				*	*
Felson 1989	*	*	*	*	*	*		*
Felson 1997	*		*	*		*	*	*
Fernandez 1986	*		*			*		
Filippou 2013	*		*	*		*	*	*
Gordon 1984	*		*	*				*
Hamza 1992	*	*	*	*	*	*		*
Hernbord 1977	*	*		*	*	*	*	*
Huang 1993	*		*	*		*		*
Latourte 2020	*	*	*	*	*	*	*	*
Ledingham 1993	*	*	*	*	*	*		*
Ledingham 1995	*	*	*	*	*	*	*	*
Massardo 1989	*	*	*	*	*		*	*
Neogi 2006	*	*		*	*	*	*	*
Neogi 2006	*	*		*	*	*	*	*
Paalanen 2020	*	*	*		*		*	*
Schouten 1992	*	*	*	*	*		*	*
Trentham 1975	*		*	*		*		*
Utsinger 1975			*	*		*		*
Van der Korst 1974	*		*	*				*

Legend: the asterisk (*) indicates the item's fulfilment

Newcastle Ottawa Scale (NOS)-Case-Control Studies

	Selection				*Comparability*	*Exposure*		
**Study**	*Case definition*	*Representativeness of the cases*	*Selection of controls*	*Definition of controls*	*Comparability of cases and controls*	*Ascertainment of exposure*	*Same method of ascertainment for cases and controls*	*Non response rate*
Bjelle 1982	*	*		*	*	*	*	*
Brasseur 1987	*	*	*	*	*		*	
Chaisson 1996	*	*	*	*	*	*	*	
Doherty 1996	*	*				*	*	
Doherty 1982	*					*	*	
Ellman 1981	*					*	*	
Ellman 1975	*		*	*	*	*	*	
Filippou 2020	*	*	*	*	*	*	*	
Good 1967	*	*	*	*	*	*	*	
Komatireddy 1989	*	*	*	*	*		*	
Ledingham 1992	*	*	*	*	*	*	*	
Ledingham 1993	*	*	*	*	*	*	*	
Mathews 1987	*	*	*	*	*		*	
McAlindon 1996	*	*	*	*	*	*	*	
Menerey 1988	*	*			*	*	*	
Montgomery 1998	*	*		*		*	*	
Musacchio 2011	*	*	*	*	*		*	
Neame 2003	*	*	*	*	*		*	
Nguyen 2013	*	*				*	*	
Pego-Reigosa 2005	*	*	*	*	*	*	*	
Pritchard 1977	*	*	*	*	*	*	*	
Reginato 1976	*		*	*		*	*	
Resnick 1977	*		*	*	*	*	*	
Resnick 1974	*	*				*	*	
Richette 2007	*	*	*	*	*	*	*	*
Sanmarti 1993	*	*				*	*	
Schlesinger 2009	*	*	*	*	*	*	*	
Stockman 1980	*	*	*		*	*	*	*
Viriyavejkul 2007	*	*	*	*	*	*	*	*
Wilkins 1983	*	*	*	*	*	*	*	*
Yashiro 1991	*	*	*	*	*	*	*	
Zhang 2004	*	*	*	*	*	*	*	*

QUADAS-2 tool- Diagnostic studies

**Study**	*Patient selection*	*Index test*	*Reference standard*	*Flow and timing*	*General assessment of RoB*
Barskova 2013					
Devauchelle-Pensec 2006					
Cipolletta 2020					
Di Matteo 2019					
Di Matteo 2017					
Ellabban 2012					
Falsetti 2011					
Falsetti 2004					
Filippou 2007					
Filippou 2016					
Filippucci 2009					
Foldes 2002					
Forien 2017					
Frediani 2005					
Gerster 1977					
Gutierrez 2014					
Lee 2019					
Ottaviani 2015					
Parperis 2013					
Ruta 2016					
Tedeschi 2020					
Vele 2018					
Zufferey 2015					

Low risk of Bias   Unclear risk of Bias   High risk of bias
